# Evolutionary and functional divergence of Sfx, a plasmid-encoded H-NS homolog, underlies the regulation of IncX plasmid conjugation

**DOI:** 10.1128/mbio.02089-24

**Published:** 2024-12-23

**Authors:** Avril Wang, Martha Cordova, William Wiley Navarre

**Affiliations:** 1 Department of Molecular Genetics, University of Toronto 7938 , Toronto, Ontario, Canada; University of Utah, Salt Lake City, Utah, USA

**Keywords:** plasmids, xenogeneic silencing, gene regulation, gene expression, conjugation, evolution

## Abstract

Conjugative plasmids are widespread among prokaryotes, highlighting their evolutionary success. Conjugation systems on most natural plasmids are repressed by default. The negative regulation of F-plasmid conjugation is partially mediated by the chromosomal nucleoid-structuring protein (H-NS). Recent bioinformatic analyses have revealed that plasmid-encoded H-NS homologs are widespread and exhibit high sequence diversity. However, the functional roles of most of these homologs and the selective forces driving their phylogenetic diversification remain unclear. In this study, we characterized the functionality and evolution of Sfx, a H-NS homolog encoded by the model IncX2 plasmid R6K. We demonstrate that Sfx, but not chromosomal H-NS, can repress R6K conjugation. Notably, we find evidence of positive selection acting on the ancestral Sfx lineage. Positively selected sites are located in the dimerization, oligomerization, and DNA-binding interfaces, many of which contribute to R6K repression activity—indicating that adaptive evolution drove the functional divergence of Sfx. We additionally show that Sfx can physically interact with various chromosomally encoded proteins, including H-NS, StpA, and Hha. Hha enhances the ability of Sfx to regulate R6K conjugation, suggesting that Sfx retained functionally important interactions with chromosomal silencing proteins. Surprisingly, the loss of Sfx does not negatively affect the stability or dissemination of R6K in laboratory conditions, reflecting the complexity of selective pressures favoring conjugation repression. Overall, our study sheds light on the functional and evolutionary divergence of a plasmid-borne H-NS-like protein, highlighting how these loosely specific DNA-binding proteins evolved to specifically regulate different plasmid functions.

**IMPORTANCE:**

Conjugative plasmids play a crucial role in spreading antimicrobial resistance and virulence genes. Most natural conjugative plasmids conjugate only under specific conditions. Therefore, studying the molecular mechanisms underlying conjugation regulation is essential for understanding antimicrobial resistance and pathogen evolution. In this study, we characterized the conjugation regulation of the model IncX plasmid R6K. We discovered that Sfx, a H-NS homolog carried by the plasmid, represses conjugation. Molecular evolutionary analyses combined with gain-of-function experiments indicate that positive selection underlies the conjugation repression activity of Sfx. Additionally, we demonstrate that the loss of Sfx does not adversely affect R6K maintenance under laboratory conditions, suggesting additional selective forces favoring Sfx carriage. Overall, this work underscores the impact of protein diversification on plasmid biology, enhancing our understanding of how molecular evolution affects broader plasmid ecology.

## INTRODUCTION

Plasmids are extrachromosomal elements found across all domains of life (1–3) and can be transferred between even phylogenetically distant organisms (4). Conjugation is a major vehicle for plasmid spread, with roughly half or more fully sequenced plasmids being conjugative or mobilizable (5, 6). However, most natural plasmids are conjugatively repressed (7), conjugating only with specific environmental cues or in the presence of viable recipient cells (8). Previous studies have suggested that limiting conjugation can (i) offset the fitness cost of plasmid carriage by reducing energy consumption (9), (ii) minimize the stress response from the expression of conjugation-related genes (10, 11), and (iii) evade bacteriophages that attach to the conjugation pilus (12–15). Therefore, a central question in plasmid biology has been to understand the molecular mechanisms that govern conjugation repression.

Plasmid conjugation in Gram-negative bacteria depends on genes encoding for components involved in mating pair formation (Mpf) and DNA transfer and replication (Dtr) (16). The Mpf genes encode the type-IV secretion system (T4SS) responsible for plasmid transfer. At the same time, the Dtr genes encode proteins that facilitate this process by nicking and coupling the plasmid origin of transfer (*oriT*) to the T4SS (16). Conjugation repression can occur by regulating the expression and/or activity of Dtr and Mpf genes (17–23), and much of these mechanisms are gleaned from studying the model F plasmid. Repression of F plasmid conjugation involves chromosomal and plasmid-encoded factors that control gene transcription (24), translation (25), and protein stability (25, 26). One such chromosomal factor is the histone-like nucleoid structuring protein (H-NS). This DNA-binding protein preferably oligomerizes along curved, AT-rich DNA, causing DNA condensation and transcriptional silencing (27, 28). Within the F plasmid, H-NS binds to promoters of conjugation-related genes (P_Y_, P_M_, and P_J_) to repress conjugation (24, 29).

Although chromosomal H-NS regulates F plasmid conjugation, many conjugative plasmids encode their own H-NS homologs. These homologs are found across different plasmid incompatibility groups, ranging from IncA/C (30), IncH (31, 32), to IncX (33, 34), indicating a selective advantage for this alternative mode of H-NS carriage. Only a few plasmid-encoded H-NS homologs have been experimentally characterized. Sfh carried by the IncH plasmid pSfR27 is functionally redundant with chromosomal H-NS and is thought to serve as a “molecular H-NS backup (31).” Other homologs are functionally unique. Acr2 carried by the IncA/C plasmid pAR060302 specifically regulates plasmid conjugation genes without affecting most chromosomal gene expression (30). H-NS_R27_ encoded by the IncH R27 plasmid also represses conjugation and is uniquely resistant to negative regulation that typically antagonizes chromosomal H-NS (32). Across plasmid-encoded H-NS homologs, those carried by IncX plasmids (i.e., the HppX clade) are notable for their sequence diversity, especially within the typically conserved DNA-binding domain (35). Although some homologs within the HppX clade are known to repress conjugation (33, 34), little is known about the processes that drove their phylogenetic diversification, the molecular mechanism of conjugation repression, their interaction with chromosomal-encoded H-NS homologs, and their broader impact on plasmid ecology.

Much of our knowledge of how H-NS regulates plasmid gene expression is gleaned from studying the F plasmid and, more recently, Sfh. However, it is unclear whether these insights are broadly applicable, especially for the phylogenetically divergent HppX clade. To address this, we characterized the evolution and function of Sfx, a H-NS homolog encoded by the model IncX2 plasmid R6K. Here, we report that the evolutionary divergence of Sfx is associated with its functional divergence from chromosomal H-NS. We find that Sfx homologs are often carried by plasmids with the MPF_T_-type T4SS and exhibit marked amino acid differences relative to chromosomal H-NS, many of which are driven by positive selection. These evolutionary signatures are accompanied by functional divergence, whereby only Sfx, but not H-NS and StpA, can repress R6K conjugation. We find that the C-terminal DNA-binding domain of Sfx is critical for its unique conjugation repression activity. We further show that Sfx can physically interact with chromosomal silencing factors, but only its interaction with Hha contributes to conjugation repression. Additionally, we find that Sfx loss minimally impacts plasmid carrier growth rate and plasmid maintenance in laboratory culture. Collectively, our work characterizes the unique evolutionary and functional divergence of a plasmid-encoded H-NS homolog, highlighting the molecular innovation afforded by protein evolution across different genomic contexts.

## RESULTS

### Sfx is associated with mobile genetic elements and diverges from other H-NS homologs

**Fig 1:**
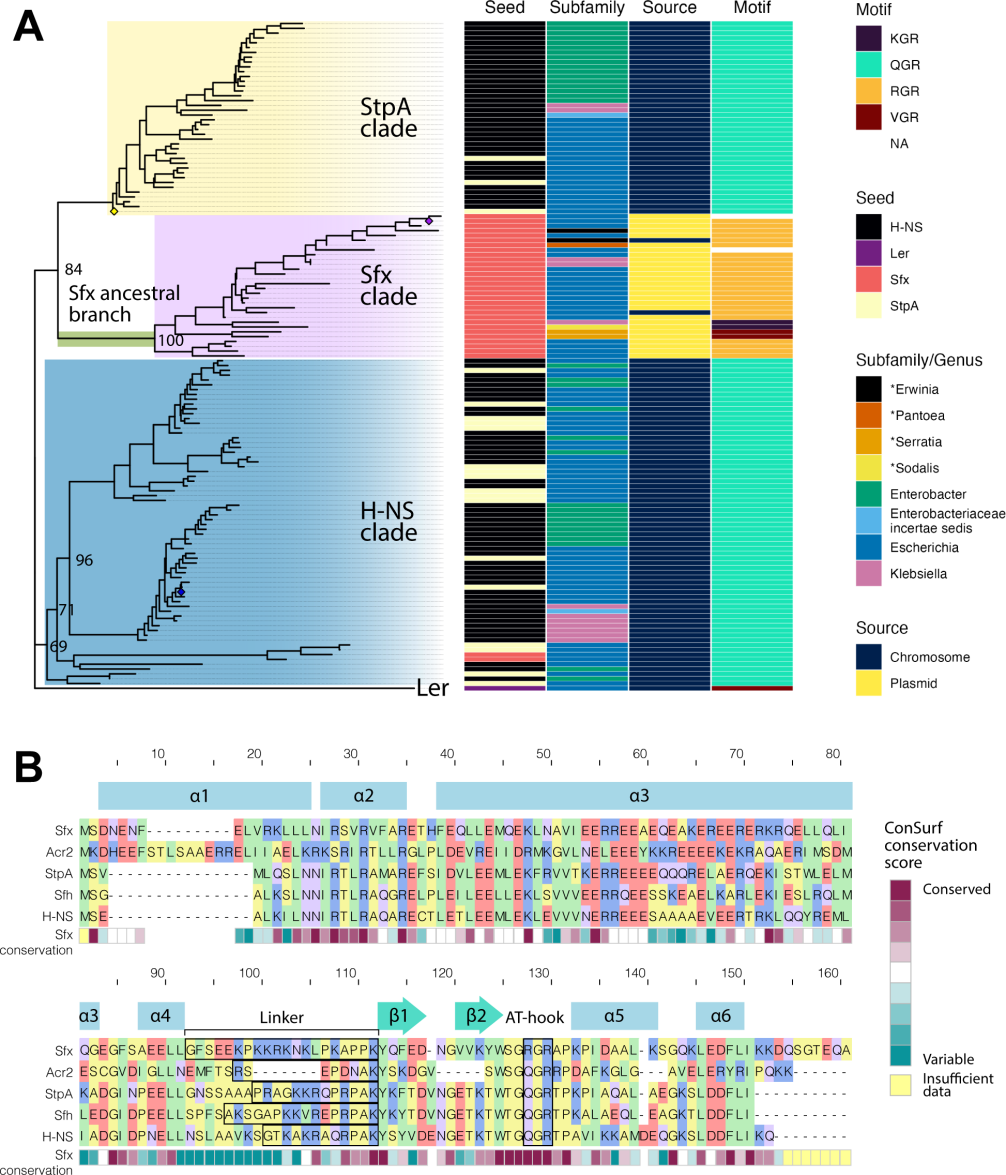
Sfx diverges phylogenetically from chromosomal and other plasmid-encoded H-NS homologs. (**A**) Annotated maximum likelihood phylogeny of Sfx, H-NS, and StpA homologs. Nucleotide coding sequences of the homologs are retrieved using tblastn and an in-house script. The sequences are aligned using codon-based alignment via Guidance2, and IQ-TREE2 is used to construct a maximum likelihood phylogeny. The Ler protein carried by *E. coli* O157:H7 str. Sakai, a distantly related H-NS-like protein, is used as the outgroup to root the phylogeny. Each sequence representative is annotated with associated characteristics (Seed = template used in tblastn, Subfamily = taxonomic classification of the bacteria encoding for the protein, Source = whether the protein is located on a chromosome or plasmid, and Motif = amino acid sequence of the AT-hook motif). Genera that begin with “*” do not belong to the *Enterobacteriaceae* family. The locations of Sfx, *E. coli* K12 H-NS, and *E. coli* K12 StpA are indicated by purple, blue, or yellow diamonds, respectively. The choice of foregrounds used in the clade models is indicated and colored (StpA = yellow, Sfx = purple, and H-NS = blue). The ancestral Sfx branch set as the foreground in the Branch-site model is highlighted in green. The displayed node values represent the ultrafast bootstrap approximates (%). (**B**) Sfx differs from other H-NS homologs in key residue positions. Amino acid sequences of Sfx (NCBI accession ID: WP_001282381.1), Acr2 (NCBI accession ID: WP_000651490.1), StpA (Uniprot accession: P0ACG1), Sfh (Uniprot accession: Q8GKU0), and H-NS (Uniprot accession: P0ACF8) are aligned using MAFFT G-INS-i. The secondary structure of Sfx, derived using POLYVIEW-2D of a ColabFold predicted structure, is indicated above the alignment, with α representing alpha-helices, β representing beta-sheets, and empty spaces representing coils. The degree of conservation at each residue position amongst Sfx homologs is calculated based on ConSurf analysis using default parameters. For each protein, the position of the linker, a disordered region after the fourth alpha-helix, is outlined with a black border.

IncX plasmids are characterized by a highly syntenic backbone, which includes a module where *sfx* is encoded immediately downstream of *topB* (DNA topoisomerase III) (36, 37). We first sought to further characterize the genomic context of Sfx homologs and their evolutionary relationships with other H-NS homologs. We queried the NCBI nr/nt database using three seed templates: Sfx, H-NS of *Escherichia coli* K12, and StpA of *E. coli* K12. This search identified 33, 86, and 21 representative sequences using the Sfx, H-NS, and StpA seed templates, respectively (Table S1). These sequences were then aligned and used for maximum likelihood phylogenetic inference. The resultant phylogeny is divided into three main clades. Two clades consist primarily of sequences obtained using H-NS and StpA as seed templates. The third clade is composed exclusively of sequences obtained using the Sfx seed template (Fig. 1A) and forms the sister group of the clade containing *E. coli* K12 StpA, consistent with previous phylogenetic analyses (35). For clarity, we designated the cluster of Sfx homologs as the Sfx clade, the group containing *E. coli* K12 H-NS as the H-NS clade, and the group containing *E. coli* K12 StpA as the StpA clade.

Sequences from the H-NS and StpA clades are all chromosomally encoded and restricted to the *Enterobacteriaceae* family (Fig. 1A). In contrast, sequences within the Sfx clade are either encoded by a plasmid or are found within chromosomal transposons (NCBI accession and range: CP013970.1:2715190–2715642 and CP028735.1:4153062–4153523). Consistent with its association with mobile genetic elements, Sfx homologs are found across a more extensive taxonomic distribution compared with proteins within the H-NS and StpA clades, including species from the *Erwinia, Pantoea, Serratia,* and *Sodalis* genera (Fig. 1A).

Despite its phylogenetic clustering, the Sfx and StpA clades differ in their AT-hook motif, a region that inserts into the minor groove of AT-rich DNA and is critical for DNA binding (38). The H-NS and StpA clades and previously characterized plasmid-encoded H-NS homologs, such as Acr2 and Sfh, carry a QGR AT-hook motif (Fig. 1A and B). In contrast, most Sfx homologs carry an RGR motif that is characteristic of xenogeneic silencing proteins encoded by bacteria with a higher genome GC content, including Lsr2 from *Mycobacteria* (39) and Bv3F from *Burkholderia vietnamiensis* (40). Another distinct property of Sfx is its longer and more proline-rich predicted linker (four prolines in Sfx linker vs. 1–3 prolines in other H-NS-like proteins; Fig. 1B), which could increase coil rigidity, limit the formation of larger nucleoprotein complexes, and increase target selectivity (41). Collectively, our findings indicate that Sfx homologs form a phylogenetically distinct clade associated with mobile genetic elements. This evolutionary divergence is partly driven by differences in residues that could impact protein oligomerization and DNA binding.

### Sfx homologs are often carried by plasmids with the MPF_T_ T4SS

Given the association between Sfx homologs and plasmids, we next explored its distribution across 55 representative IncX plasmid lineages (Table S2). Despite significant sequence divergence among the plasmid lineages—with no identifiable core ORFs according to Roary (42)—a little more than half of the plasmids (31/55; 56.4%) encode for H-NS homologs. Most of these plasmids carry a H-NS homolog with the RGR AT-hook motif (25/31; 80.6%) characteristic of the Sfx clade (Fig. S1). We next assessed whether plasmids carrying Sfx and H-NS homologs differ in the encoded MPF system. The T4SSs of *Proteobacterial* conjugative plasmids are broadly classified into four MPF classes based on protein homology: MPF_F_, MPF_I_, MPF_G_, and MPF_T_ (43). IncX plasmids often carry MPF_T_ T4SS (44), which tends to confer only the capacity to mate on solid surfaces (43). We find that IncX plasmids encoding for a H-NS homolog tend to also carry the MPF_T_ T4SS (Fig. S1). Conversely, plasmids lacking a MPF system or possessing a MPF_F_ T4SS (i.e., the type of T4SS possessed by the F plasmid) tend not to carry Sfx or any other H-NS homolog (Fig. S1). Taken together, our findings reaffirm the conservation of Sfx across IncX plasmids and reveal a close association of Sfx with MPF_T_ T4SS, suggesting that Sfx may act as a regulator uniquely specific for MPF_T_-type conjugation systems.

### Positive selection drove the evolutionary divergence of Sfx

Next, we used molecular evolution analysis to investigate the evolutionary processes that drove the divergence of the Sfx clade. We first employed clade models to determine the selective forces affecting extant sequences. Our best-performing model (CmD with Sfx and StpA clades set as separate foregrounds) suggests that most protein sites of extant H-NS, StpA, and Sfx clades show signatures of purifying selection (Table S3; Fig. S2), indicating their functional importance. Notably, the StpA clade exhibits the least degree of selective constraints (Fig. S2), which may reflect its recessive phenotype and indicate its potential for adaptive evolution.

We next used the branch-site test to determine the evolutionary processes that drove the divergence of the ancestral Sfx lineage from the StpA clade (ancestral branch highlighted in green in Fig. 1A). The branch-site test detects signatures of positive selection from a single branch selected *a priori* (the foreground) within a phylogeny (the background). Adaptive evolution/positive selection can be inferred if fitting an alternative branch-site model incorporating positive selection leads to a statistically significant improvement in model fit relative to fitting a null model, where only neutral and purifying evolution is considered (45). We find that fitting the alternative branch-site model leads to a statistically significant (*P*-value = 0.015) improvement in model fit relative to the null model (Table S3), suggesting that positive selection occurred after the Sfx clade diverged from ancestral StpA.

**Fig 2:**
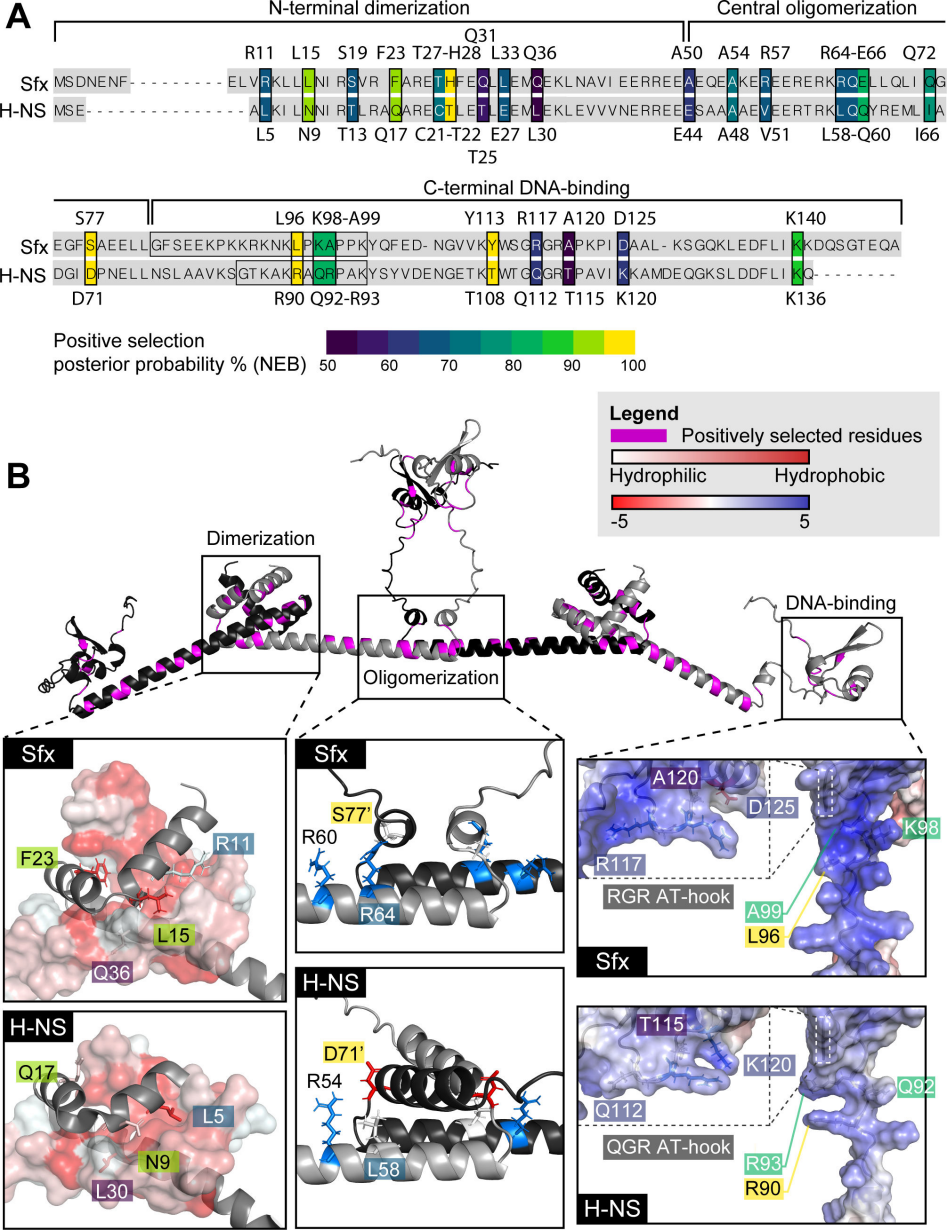
Positively selected sites map to Sfx dimerization, oligomerization, and DNA-binding interfaces. (**A**) Positions of positively selected sites mapped onto an alignment of Sfx (R6K) and *E. coli* K12 H-NS. The identity and position of each positively selected site are displayed above (Sfx)/below (H-NS) the alignment. Each site is colored based on the posterior probability that the site has experienced positive selection following the divergence of the ancestral Sfx lineage (naive empirical Bayes results). The linker region of each protein is outlined with a black box. (**B**) Positively selected sites mapped onto the predicted structures of Sfx and H-NS. The top panel displays the ColabFold-predicted tetrameric structure of Sfx. Residues that have undergone positive selection are colored in magenta. The interaction interfaces of the dimerization, oligomerization, and DNA-binding domains are displayed in the inset boxes, with the top panel containing the predicted Sfx structure and the bottom panel containing the predicted H-NS structure. The N-terminal dimerization domain inset displays the interaction between a dimer (one as a grey cartoon structure and the other as a surface representation structure). The hydrophobicity of the surface representation structure is indicated by its color, and positively selected residues that form the hydrophobic interaction interface of H-NS are labeled. The central oligomerization domain inset displays the interaction between two dimers (one colored in gray and the other colored in black). The locations and charge (blue = positively charged, red = negatively charged) of positively selected residues participating in H-NS oligomerization are labeled. The apostrophe indicates that the residue is located on another protein. Although not positively selected, R60/R54 (Sfx/H-NS) is highlighted due to its ionic interaction with D71 in H-NS. The DNA-binding domain inset displays a surface representation of the linker region and the AT-hook motif (highlighted by a dashed box) colored based on its electrostatic charge (blue = positively charged, red = negatively charged). The locations of positively selected residues that participate in DNA binding in H-NS are labeled.

We next analyzed the potential functionality of positively selected residues indicated by the naive empirical Bayes (NEB) and Bayes empirical Bayes (BEB) analyses (see Table 1 for details). Although NEB outputs are more prone to false positives than BEB for small data sets (46), both methods produced similar results (Fig. 2A; Table 1). Most positively selected sites are conserved within the Sfx, H-NS, and StpA clades (Fig. S3), indicating their functional importance. Overall, positive selection altered the dimerization and oligomerization interfaces of Sfx relative to H-NS (Fig. 2B), which may change the protein’s nucleation profile and heteromeric interactions. For instance, the N9L mutation (corresponding to the L15 (Sfx)-N9 (H-NS) transition) reduces H-NS’ capacity to interact with Hha and repress the hemolysin (*hly*) operon (47), indicating that Sfx may display altered interaction with Hha. Although not directly part of the dimerization interface, the T27-C21 transition is biochemically similar to the C21S mutation in H-NS, also known to decrease DNA-binding affinity (48). Changes to the oligomerization domain may also impact protein regulation. H-NS is temperature-sensitive, with higher temperature destabilizing the oligomerization interface (e.g., by disrupting the ionic bond D71:R54') (49, 50), causing H-NS to adopt an inhibited configuration (49) and relieve repression (51–53). In Sfx, positive selection led to divergence in many of the sites involved in H-NS dimer-dimer interaction and H-NS auto-inhibition (e.g., S77-D71; Fig. 2B; Table 1) (49, 50).

**TABLE 1:** Characteristics of positively selected residues defined based on NEB and BEB analyses^*a*^

Domain	Sfx residue	H-NS residue	NEB	BEB	Functional roles
N-terminal dimerization	R11	L5	Positively selected	Positively selected	Hydrophobic interactions by L5 in H-NS stabilize dimer formation *in silico* (50).
N-terminal dimerization	L15	N9	Positively selected	Positively selected	N9L mutation in H-NS disrupts H-NS-Hha interaction, increases tendency to form oligomers in the presence of DNA, and decreases its capacity to the repress the *hly* operon (47, 54).
N-terminal dimerization	S19	T13	Positively selected	Positively selected	Phosphorylation of T13 in H-NS (*Salmonella enterica*) disrupts H-NS dimer formation and decreases binding and repression of PhoP/PhoQ-dependent promoter regions (55).
N-terminal dimerization	F23	Q17	Positively selected	Positively selected	Q17P mutation of H-NS (*S. enterica*) disrupts alpha-helix two and oligomerization (56). In the C21S H-NS mutant, molecular dynamics simulations indicate that S21 forms a hydrogen bond with Q17 that stabilizes the parallel complex conformation (57) — a finding contested by small angle X-ray scattering experiments showing that the C21S H-NS mutant maintains the anti-parallel complex conformation (49).
N-terminal dimerization	T27*	C21	Positively selected	Positively selected	C21F H-NS mutant has similar stability to wildtype H-NS but the F21C StpA mutant are more likely to form dimers and are less susceptible to proteolysis by Lon (58). C21S H-NS (*S. enterica*) mutant has reduced binding affinity to *slp* promoters *in vitro* (48). C21S mutation in H-NS allows hydrogen bond formation between S21 and protein backbone, promoting parallel complex formation (57).
N-terminal dimerization	H28	T22	Positively selected	Positively selected	—
N-terminal dimerization	Q31	T25	Positively selected	Negatively selected	—
N-terminal dimerization	L33	E27	Positively selected	Positively selected	E27 is involved in intramolecular interaction between H-NS N-terminus and C-terminus that promotes autoinhibition (49).
N-terminal dimerization	Q36	L30	Positively selected	Negatively selected	L30P and L30D (but not L30A or L30K) H-NS mutants abrogates dimer formation and *proV* repression (59). Additionally, L30P H-NS mutant cannot repress *hdeA* and *hchA* transcription (60). L30 is involved in intramolecular interaction between H-NS N-terminus and C-terminus that promotes autoinhibition (12). L30P mutation of *Vibrio cholerae* H-NS (69% similarity to *E. coli* H-NS) does not affect its ability to dimerize nor repress *ctx–lacZ* and *toxT–lacZ* lysogens (61).
Central oligomerization	A50	E44	Positively selected	Negatively selected	E44 is involved in intramolecular interaction between H-NS N-terminus and C-terminus that promotes autoinhibition) (49, 50).
Central oligomerization	A54	A48	Positively selected	Positively selected	—
Central oligomerization	R57	V51	Positively selected	Positively selected	—
Central oligomerization	R64	L58	Positively selected	Positively selected	L58 is part of the hydrophobic core that stabilizes H-NS oligomerization (27)
Central oligomerization	Q65	Q59	Positively selected	Positively selected	—
Central oligomerization	E66	Q60	Positively selected	Positively selected	—
Central oligomerization	Q72	I66	Positively selected	Positively selected	—
Central oligomerization	S77*	D71	Positively selected	Positively selected	Molecular dynamics simulations suggest that D71 forms intramolecular ionic bonds with R90 and intermolecular ionic bonds with R54 (49). A D71A mutation (which breaks the ionic bonds) decreases the extent to which increasing salinity disrupts the central oligomerization domain (49). H-NS mutant unable to form the R54-D71 salt bridge (R54M) have reduced capacity to oligomerize and are less sensitive to temperature and salinity (50). H-NS double mutant (D68V, D71V) have higher tendency to oligomerize *in vitro* (62).*7/2/2024 4:52:00 PM*
Linker	L96*	R90	Positively selected	Positively selected	Molecular dynamics simulations suggest that D71 forms intramolecular ionic bonds with R90 and intermolecular ionic bonds with R54 (49). Disruption of R90-D71 decreases the extent to which increasing salinity disrupts the central oligomerization domain (49). R90 is involved in DNA-binding *in vitro* (41, 63) and is shown, via molecular dynamics simulations, to insert into the DNA minor groove (64). R90G mutation decreases H-NS DNA binding affinity *in vitro* (41). R90H/C mutation derepresses *proV* expression (65).
Linker	K98	Q92	Positively selected	Positively selected	Q92 is involved in DNA-binding *in vitro* (63).
Linker	A99	R93	Positively selected	Positively selected	R93 is involved in DNA binding *in vitro* (63, 66), while the R93G mutation decreases H-NS DNA binding affinity *in vitro* (41, 67). R93C/H mutations derepress *proV, proU,* and *fimB* expression (65, 67). R93 is involved in intramolecular interaction between H-NS N-terminus and C-terminus that promotes autoinhibition (49, 50).
C-terminal DNA-binding	Y113**	T108	Positively selected	Positively selected	T108I mutation derepresses *proU* and *fimB* expression and decreases binding affinity to *fimB* promoter *in vitro* (67). However, the T108I mutant can still compensate for reduced motility of *hns* mutant and regulate *fimA* promoter inversion rate (67). T108 is involved in intramolecular interaction between H-NS N-terminus and C-terminus that promotes autoinhibition (49).7/2/2024 4:52:00 PM
C-terminal DNA-binding	R117	Q112	Positively selected	Negatively selected	Q112 inserts into the DNA minor groove (66). Q112A reduced H-NS DNA-binding affinity *in vitro* (66).
C-terminal DNA-binding	A120	T115	Positively selected	Negatively selected	T115 is directly involved in DNA-binding *in vitro* (63), while the T115I mutation derepresses *proV* expression (65). T115 is predicted *in-silico* to make close contact with c-di-GMP, a putative co-factor of H-NS. T115A mutation decreases ci-di-GMP affinity to H-NS *in vitro* (68).
C-terminal DNA-binding	D125	K120	Positively selected	Positively selected	K120 interacts with DNA *in-silico* and *in vivo* (50, 69). K120 can undergo succinylation and acetylation (70). K120 can undergo 2-hydroxyisobutyrylation (71).
C-terminal DNA-binding	K140	K136	Positively selected	Positively selected	K136 can undergo succinylation and acetylation (70). K136 can undergo 2-hydroxyisobutyrylation (71).

^*a*^ This table lists all residue positions that show evidence of positive selection within the ancestral Sfx branch, but not among the background sequences (Background ω = 0.105 and Sfx ω = 11.423) using BEB or NEB. Residue positions with a > 95% posterior probability of being positively selected via NEB are indicated by * or ** if both NEB and BEB indicate a posterior probability of being positively selected >95%. Each position in the protein sequence alignment is assigned to domains according to Zhao et al. (50), with the corresponding residues in Sfx and H-NS listed in columns 2 and 3, respectively. Functional annotations are derived from a comprehensive literature review H-NS of *E. coli* and *S. enterica*.

Among H-NS homologs, only the HppX clade significantly deviates from chromosomal H-NS at the DNA-binding domain (35). Our data suggest that positive selection drove the transitions of key residues that directly interact with DNA in H-NS (Fig. 2B; Table 1), many of which are positively charged. Interestingly, we find that positive selection underlies the unique RGR AT-hook motif of Sfx (Fig. 2B; only under positive selection by NEB but not BEB), further supporting its potential functional significance. Together, our results suggest that the ancestral Sfx lineage experienced positive selection at numerous sites, many of which correspond to residues implicated in protein-protein interaction, environmental regulation, and DNA binding.

### Sfx, but not H-NS and StpA, represses R6K conjugation

**Fig 3:**
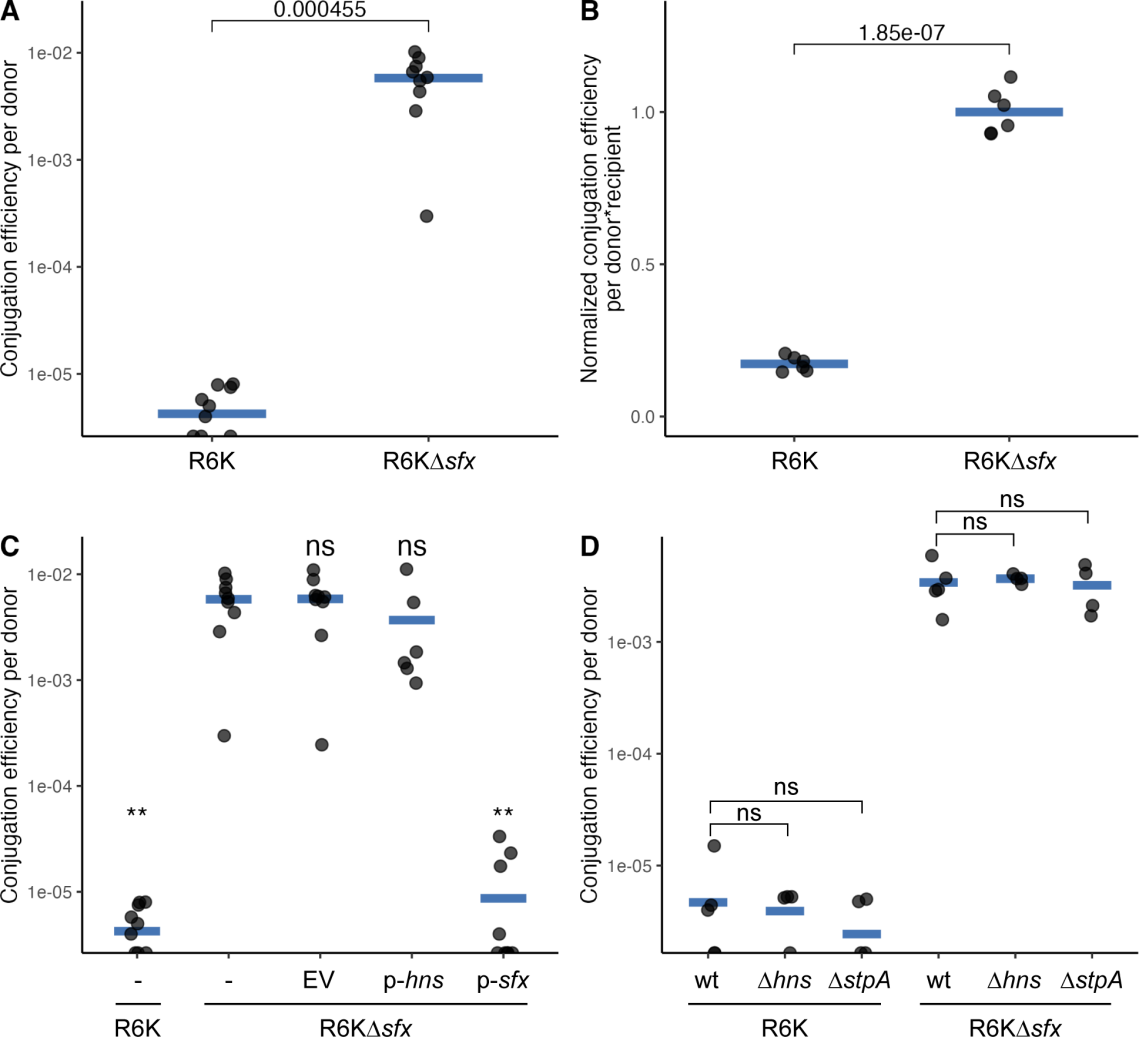
Sfx represses R6K conjugation on filter paper and within biofilms. (**A**) *sfx* deletion increases R6K conjugation on filter-paper. Two OD_600_U of donor (*E. coli* K12 carrying R6K or R6K∆*sfx*) and recipient cells (NaN_3_^r^ EcoR25) are incubated on a filter-paper disc at 30˚C for 3 h. Conjugation efficiency is measured by the number of transconjugants divided by the number of donors at the end of the conjugation period. (**B**) *sfx* deletion increases R6K conjugation in biofilm. Donor (*E. coli* K12 carrying R6K or R6K∆*sfx*) and recipient cells (NaN_3_^r^ EcoR25) are seeded in M9 +0.2% glucose media to a final OD_600_ of 0.03 and grown at 30˚C for 24 h. Conjugation efficiency is assessed by dividing the number of transconjugant cells by the product of donor and recipient populations (to account for potential differences in recipient population growth). Conjugation efficiency is further normalized by scaling the mean conjugation efficiency of R6K∆*sfx* carriers to 1. (**C**) Sfx, but not H-NS, complementation decreases R6K∆*sfx* conjugation efficiency. The *y*-axis displays the conjugation efficiency of different donor cells (NaN_3_^r^ EcoR25 recipient cells) on filter paper. The *x*-axis indicates the plasmids carried by the donor cells. (**D**) Chromosomal H-NS and StpA do not repress R6K conjugation. The *y*-axis displays the conjugation efficiency of different donor cells on filter paper. The *x*-axis indicates the donor’s genotype (top row) and plasmid (bottom row). (**A–D**) The data displayed are pooled results from at least two experimental replicates, and each point represents one biological replicate (averaged value across two technical replicates). The Student’s *t*-test is used to assess statistical significance, and the Benjamini-Hochberg correction is applied when more than one *t*-test is conducted. Abbreviations: ns (*P*-value ≥ 0.05); – (placeholder); EV (pHSG576 empty vector, low-copy); p*-hns* (pHSG576-*hns,* native *hns* promoter); p-*sfx* (pHSG576-*sfx,* native *sfx* promoter).

We next examined whether the sequence divergence displayed by Sfx is of functional importance. To test this, we performed filter paper conjugation experiments involving *E. coli* carrying wild-type R6K plasmid or one lacking *sfx* (R6K∆*sfx*). Deletion of *sfx* increased R6K conjugation efficiency on filter paper by >1,000-fold (Fig. 3A). Considering that the filter paper is an artificial setting for conjugation, we examined whether Sfx loss would also increase R6K spread in a more naturalized biofilm setting (72). We find that R6K*∆sfx* conjugates at a higher efficiency than R6K in biofilms (~5-fold; Fig. 3B), affirming that Sfx is a negative regulator of plasmid transmission.

To ensure that the impact of Sfx loss is not polar, we transformed a separate low-copy-number plasmid expressing Sfx from its native promoter into donor cells. We find that complementation reduced the conjugation efficiency of R6K∆*sfx* donors to levels comparable with that of wild-type R6K donors (Fig. 3C). Interestingly, expressing chromosomal H-NS from the same low-copy plasmid backbone does not affect the conjugation efficiency of R6K∆*sfx* (Fig. 3C). To test whether chromosomal H-NS and its paralog StpA can also regulate R6K conjugation, we repeated the filter paper conjugation experiments using ∆*hns* and ∆*stpA* donors. We find that neither of these chromosomal deletions affects the conjugation efficiency of R6K and R6K∆*sfx* (Fig. 3D), indicating that chromosomal H-NS homologs do not regulate R6K conjugation. Collectively, our data suggest that Sfx represses R6K conjugation and has functionally diverged from chromosomal H-NS and StpA.

### Sfx can partially compensate for H-NS loss

**Fig 4:**
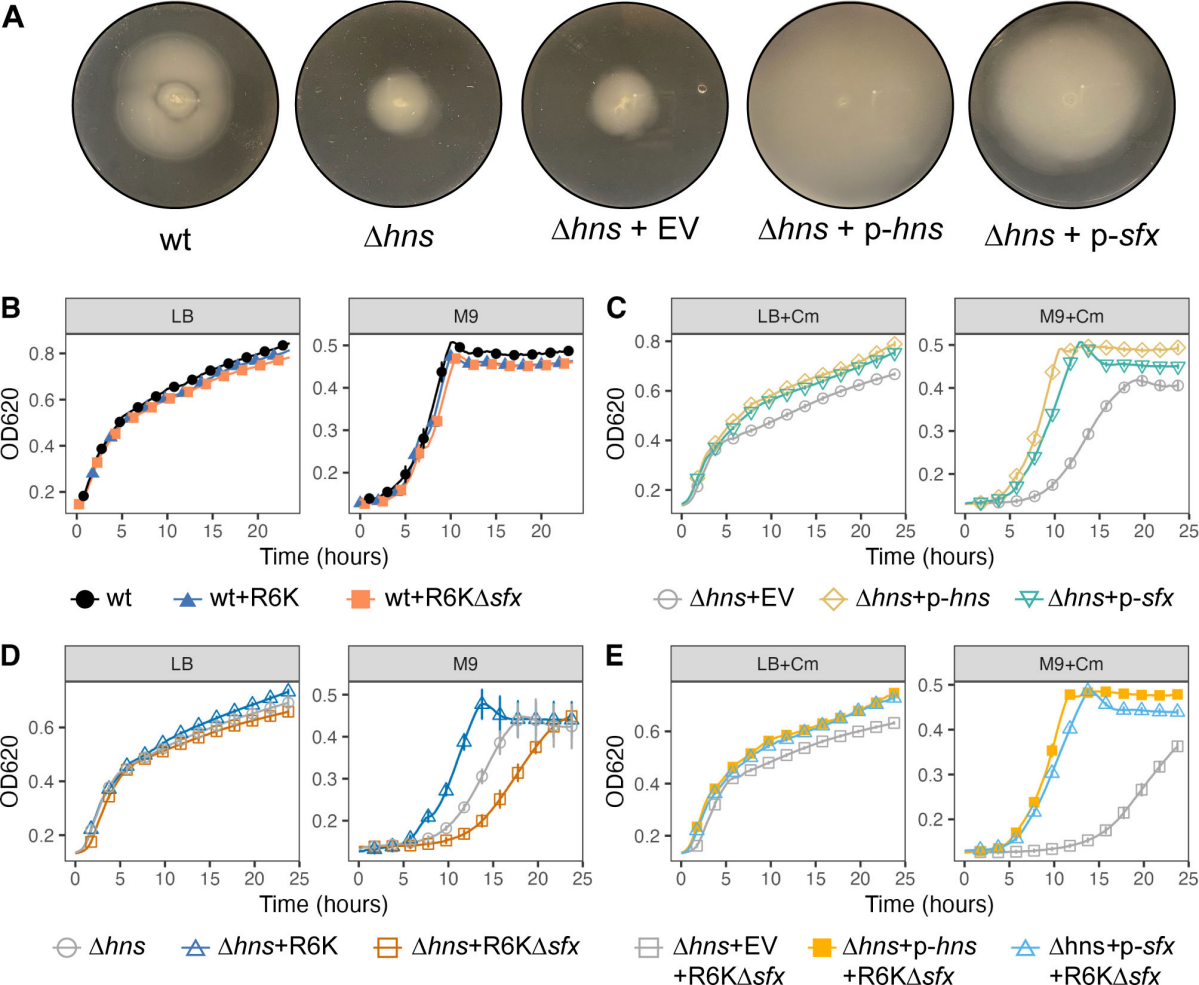
Sfx can partially compensate for ∆*hns* phenotypes. (**A**) Expressing Sfx increases the motility of *hns* mutants. Motility assays are performed by inoculating 6 µL of *E. coli* Keio Collection strains (genotype displayed below the plates) subcultured to a mid-log phase (OD_600_, ~0.6) into the center of a soft-agar plate (0.3% agar). The plates are incubated at 30˚C for 24 h. The images are representative of two independent experimental replicates, each with two biological replicates. (**B–E**) Time series growth curve data of various *E. coli* strains grown at 37˚C in LB or M9 + 0.2% glucose are displayed. When required, 20 µg/mL of chloramphenicol (“Cm”) is added to maintain stable inheritance of the pHSG576 low-copy plasmids. Each data point represents the mean ± standard deviation across two biological replicates, each with two technical replicates. The data are representative of three independent experimental replicates. Abbreviations: wt (wildtype *E. coli* BW25113); EV (pHSG576 empty vector, low-copy); p*-hns* (pHSG576-*hns,* native *hns* promoter); p-*sfx* (pHSG576-*sfx,* native *sfx* promoter).

Plasmid-encoded H-NS homologs H-NS_R27_ and Sfh can partially compensate for ∆*hns* phenotypes (73, 74). Considering H-NS’s inability to repress R6K conjugation, we wondered whether Sfx, in contrast, can rescue the null phenotypes of *E. coli*∆*hns*. The presence of R6K or a low-copy plasmid expressing Sfx from its native promoter compensates for the reduced motility and growth lag of *hns* mutants (75) (Fig. 4A and C). Conversely, although R6K∆*sfx* carriage does not significantly impact the growth dynamics of wild-type *E. coli* (Fig. 4B)*,* it reduces the growth rate of *hns* mutants (Fig. 4D), suggesting that at least one H-NS homolog (chromosomal H-NS or Sfx) is needed to mitigate the fitness burden of R6K carriage. Accordingly, expression of H-NS or Sfx from its native promoter of a low-copy plasmid rescued the growth defect exhibited by the *hns* mutant carrying R6K∆*sfx* (Fig. 4E). Overall, although H-NS cannot repress R6K conjugation, Sfx can partially compensate for H-NS function, suggesting a partial, albeit asymmetrical, overlap between the regulons of H-NS and Sfx.

### The DNA-binding domain is necessary for Sfx-mediated conjugation repression

**Fig 5:**
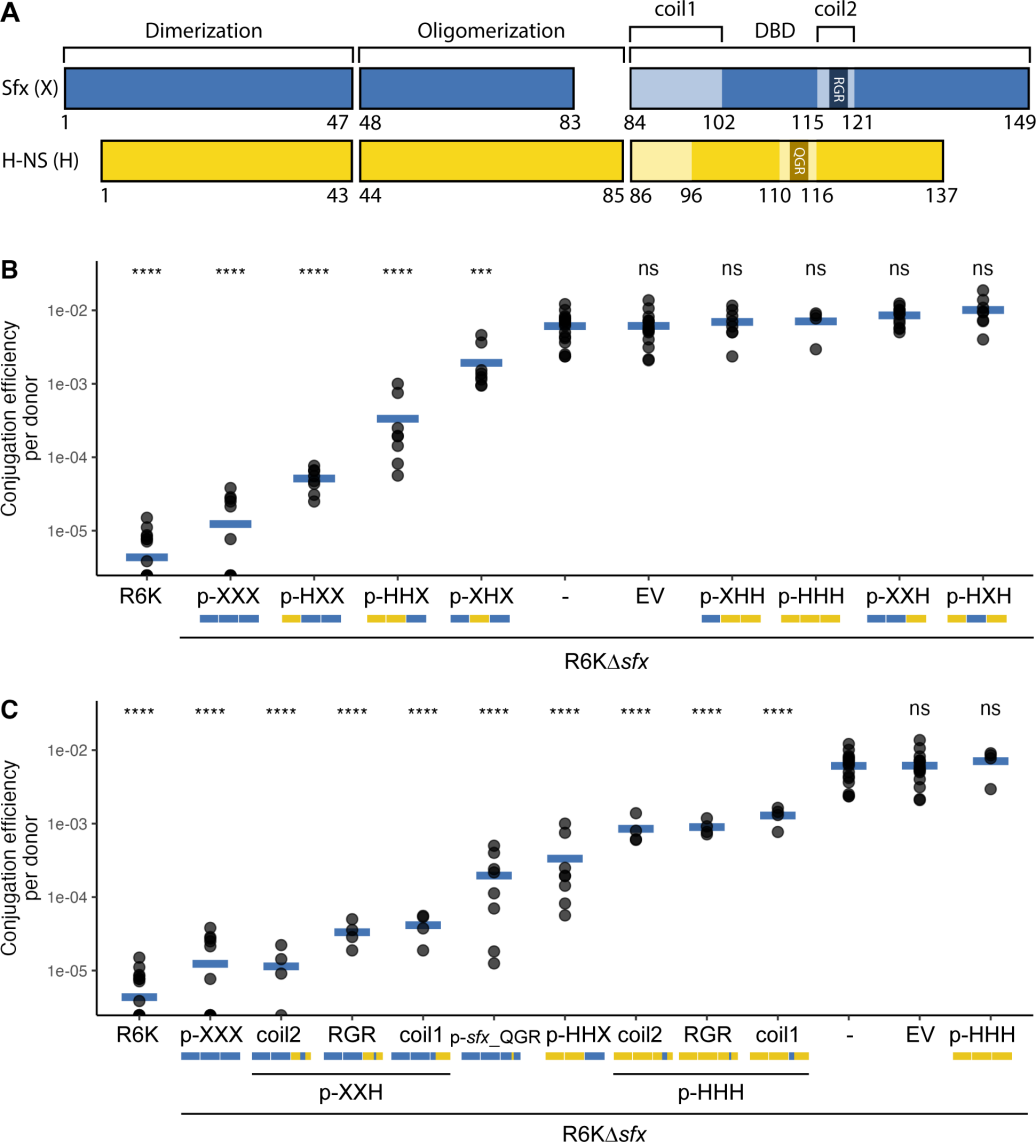
Sfx C-terminus is necessary for R6K conjugation repression. (**A**) Schematic of domain assignment for Sfx and H-NS. Secondary structure assignments are based on the ColabFold-predicted structure of Sfx and H-NS. Coil 1 (linker) and coil 2 (contains the AT-hook motif) are two unstructured regions within the DNA-binding domain (DBD). The precise amino acid position at which the domains are split (and where the chimeras are fused) is displayed below each protein schematic. (**B and C**) The results from filter paper conjugation experiments performed with various donors (indicated on the *x*-axis) and NaN_3_^r^ EcoR25 recipients are displayed. The identity of the chimeric proteins expressed from a low-copy plasmid (*sfx* promoter) is represented by p-NNN where each N represents the domain identities (X = Sfx, H = H NS) and a mini graphic schematic (blue = Sfx, yellow = H NS). (**B**) The Sfx DNA-binding domain is needed for R6K conjugation repression. The *y*-axis displays the conjugation efficiency (transconjugant population divided by donor population) of donors carrying R6K or R6K∆*sfx* and low-copy plasmids encoding for chimeric proteins. Only R6K∆*sfx* carriers that express chimeric proteins with the Sfx DNA-binding domain (p-NNX) exhibit statistically significantly reduced (*P*-value < 0.05) conjugation efficiency relative to no plasmid control (R6K∆*sfx* only). (**C**) The linker and the AT-hook motif contribute to Sfx’s conjugation repression activity. The same conjugation assay is performed as in panel **B**, albeit with donors expressing chimeric proteins with point or segmental mutations. Coil 1/2 indicates chimeric proteins carrying Sfx’s coil 1/2 (in either a p-XXH or p-HHH background), whereas p-*sfx_*QGR represents plasmid encoding for a Sfx protein with the QGR AT-hook motif. For panels **B and C**, statistical significance is assessed using the Student’s *t*-test (reference group is *E. coli* carrying only R6K∆*sfx*) with Benjamini-Hochberg correction. The data are representative results from two independent experimental replicates. Abbreviations: **** (adjusted *P*-value < 0.0001); *** (adjusted *P*-value < 0.001); ns (adjusted *P*-value ≥ 0.05).

Given that Sfx functionally diverges from H-NS, we performed a series of gain-of-function experiments to explore which structural elements confer Sfx’s unique ability to repress R6K conjugation. We constructed chimeric proteins with different combinations of H-NS and Sfx domains (indicated in Fig. 5A), expressed them from a low-copy plasmid off the native *sfx* promoter, and assessed their impact on the conjugation efficiency of R6K∆*sfx*.

All chimeras containing Sfx’s DNA-binding domain can repress conjugation of R6K∆*sfx* to varying degrees (Fig. 5B)*,* suggesting that the DNA-binding domain is important for Sfx’s repression activity. The addition of Sfx’s central oligomerization domain improves chimeric protein’s repression activity (p-HXX vs. p-HHX; Fig. 5B), which could suggest that the oligomerization domain contributes to Sfx repression only when the C-terminal domain is present and/or that the presence of the domain improves protein expression/folding.

To narrow down the regions within Sfx’s DNA-binding domain that contribute to conjugation repression, we swapped smaller segments of the H-NS DNA-binding domain with corresponding segments from Sfx. Exchanging either the linker region (coil 1) or the coiled region surrounding the AT-hook motif (coil 2) conferred partial conjugation repression to constructs containing the H-NS DNA binding domain (Fig. 5B and C), suggesting that Sfx’s linker and its unique RGR AT-hook motif are major contributors to its unique repression activity. To further confirm that the functional importance of coil 2 is derived from the RGR AT-hook motif (and not the surrounding residues), we constructed Sfx mutants that carried the QGR AT hook motif and H-NS mutants that carried the RGR AT hook motif. The RGR to QGR mutation decreased but did not completely abolish Sfx’s conjugation repression activity (p-*sfx_*QGR; Fig. 5C), suggesting that the RGR AT-hook motif and other motifs (e.g., linker) function independently to repress R6K conjugation. Strikingly, the H-NS mutant bearing a single QGR to RGR mutation can partially repress R6K∆*sfx* conjugation with an efficiency similar to that of H-NS chimeras carrying the entire Sfx coil 2 (~10-fold repression, Fig. 5C). These findings indicate that the RGR AT-hook motif alone accounts for the functional importance of coil 2. Altogether, our results provide evidence that the sequence variations displayed within the DNA-binding domain of Sfx are central to its unique conjugation repression activity.

### Sfx interacts with Hha to repress R6K conjugation

H-NS regulatory activity is modulated by its interactions with StpA (76–78), Hha (51, 78, 79), and Cnu/YdgT (80, 81). StpA is a paralog of H-NS that can interact with H-NS to form more thermally stable heterodimers (82) and bridged filaments (H-NS:StpA filaments that connect two DNA duplexes) (78, 83). These H-NS:StpA filaments promote greater RNA polymerase pausing (78) and could be important for gene regulation at high temperatures where H-NS repression is relieved (51, 52). H-NS also interacts with Hha and its paralog Cnu via its dimerization domain (84, 85). H-NS-Hha interaction promotes the formation of bridged filaments that stimulate greater RNA polymerase pausing (78) and is needed to repress expression of various virulence-related genes (e.g., *hlyCABD* operon (51, 79), LEE pathogenicity island (86, 87), *Salmonella* pathogenicity island 2 (88)). Although R6K does not carry a Hha homolog, certain conjugative plasmids do (89). For the IncH conjugative plasmid R27, plasmid-encoded H-NS and Hha participate in temperature-dependent conjugation repression (73). The role of Cnu is less understood (81), although recent *in vitro* work suggests that Cnu may be involved in osmolarity response (90).

Given the importance of protein-protein interactions for H-NS activity and the fact that plasmid-encoded H-NS, such as Sfh, is known to interact with chromosomal H-NS and StpA (91), we used a bacterial two-hybrid system to examine the physical interactions between Sfx, H-NS, StpA, Hha, and Cnu. Due to the toxicity of H-NS overexpression in the presence of ampicillin (92), we used a truncated H-NS lacking its DNA-binding domain (M1-G85) for assays involving high-copy vectors (pUT18; Amp^R^) encoding for H-NS.

**Fig 6:**
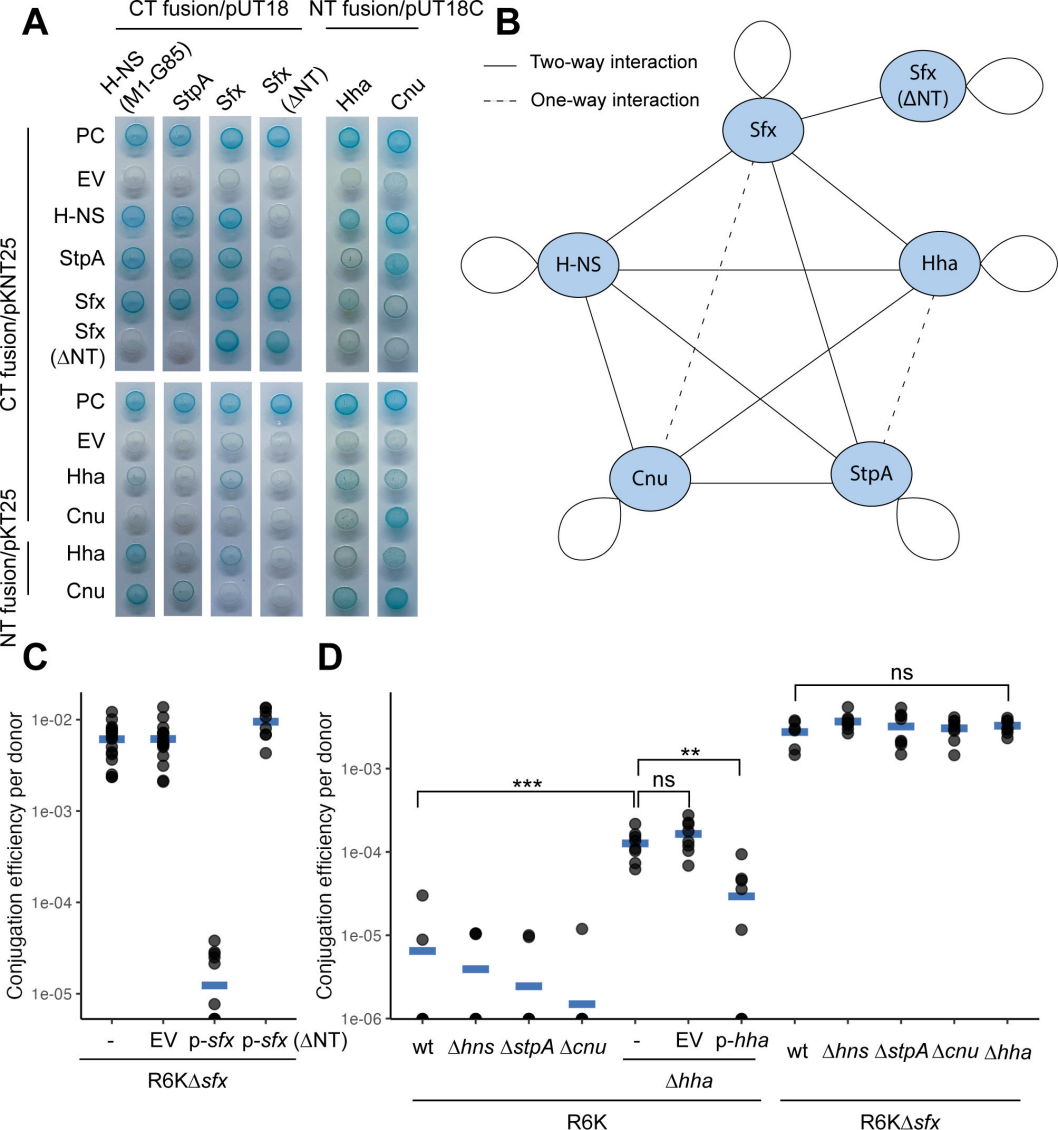
Sfx-Hha interaction contributes to conjugation repression. (**A**) Sfx interacts with H-NS, StpA, and Hha. Bacterial two-hybrid assay is used to assess binary protein-protein interactions between H-NS, Sfx, StpA, Hha, and Cnu. A blue spot indicates that the expressed fusion proteins interact with sufficient strength to reconstitute the split adenylate cyclase, leading to the expression of a cAMP-CAP-regulated *lacI*. A white spot indicates either insufficient interaction between the two Fusion proteins or insufficient expression. Fusion proteins are either made by fusing the adenylate cyclase fragment to the C-terminus (CT) or N-terminus (NT). We expressed the fusion proteins either from a high-copy (pUT18/pUT18C) or a low-copy (pKT25/pKNT25) plasmid. H-NS (M1-G85) lacking the DNA-binding domain is used in lieu of the full-length protein due to the toxicity of H-NS expression from a high-copy vector in the presence of ampicillin. Sfx (∆NT) represents an N-terminally truncated variant (∆D3-E8). Each assay is performed with a positive control (PC; *E. coli* carrying pKNT25-*hns* and pUT18-*stpA*, two proteins that are known to interact) and a negative control (*E. coli* carrying the plasmid indicated on the top row and a pKNT25/pKT25 empty vector). The assay is performed with three biological replicates, each with two technical replicates. The presented images are representative of two independent experiments. (**B**) Network depiction of bacterial two-hybrid results. The solid lines suggest that protein interaction is detected for both protein construct combinations (e.g., pUT18-*hns* and pKNT25-*stpA*, pUT18-*stpA,* and pKNT25-*hns*). The dotted lines suggest that protein interaction is detected only with one of the protein construct combinations. (**C**) N-terminally truncated Sfx cannot repress R6K conjugation. Various constructs (“–” = no plasmid, EV = empty vector, p-*sfx* = pHSG576 *sfx*, p-*sfx* (∆NT) = pHSG576 *sfx* (∆E3-D8)) are transformed into *E. coli* carrying R6K∆*sfx* and tested for their ability to repress conjugation efficiency on filter paper. (**D**) Hha co-represses R6K conjugation with Sfx. Filter paper conjugation is performed with various *E. coli* donors from the Keio collection. The genotypes of the donors are indicated by the top row in the x-axis, and the plasmids they carry are displayed in the bottom row. Complementation of *hha* deletion is performed by transforming R6K carriers with EV (pHSG576 empty vector) or p-*hha* (pHSG576-*hha* with native *hha* promoter, low-copy). For panels **C and D**, the data shown are representative of two independent experiments. When indicated, the Student’s *t*-test with Benjamini-Hochberg correction is used to assess statistical significance. Abbreviations: *** (adjusted *P*-value < 0.001); ** (adjusted *P*-value < 0.01); ns (adjusted *P*-value ≥ 0.05).

We first validated our assay by recovering previously characterized interactions, including those between H-NS and StpA (93), H-NS and Hha (94), H-NS and Cnu (80, 95), and StpA and Cnu (95) (Fig. 6A). We find that Sfx interacts with H-NS, StpA, and Hha, whereas Sfx-Cnu interaction is only detected when Cnu is expressed from a high-copy vector (pUT18/pUT18C, Fig. 6A and B). Interestingly, a fusion of the reporter fragment to the N- or C-terminus of Hha did not affect Hha-Sfx interaction, unlike Hha-H-NS interaction, which is weakened by Hha C-terminal fusion (Fig. 6A) (94). This discrepancy in Hha sensitivity to terminal fusions indicates potential differences between Sfx-Hha and H-NS-Hha interactions, which corroborates with the fact that key residues involved in H-NS-Hha interactions underwent positive selection in the ancestral Sfx lineage (e.g., L15-N9 transition).

Interestingly, Sfx that lacks its N-terminal extension (∆D3-E8/∆NT) cannot form heteromeric interactions but can still interact with wild-type Sfx and N-terminally truncated Sfx (Fig. 6A). N-terminally truncated Sfx cannot repress R6K∆*sfx* conjugation when expressed from a low-copy plasmid (Fig. 6C), suggesting that heteromeric interactions are needed for Sfx-mediated conjugation repression and/or that the N-terminal extension is necessary for proper Sfx-Sfx interaction. To explore the former possibility, we assessed whether Sfx’s interactions with H-NS, StpA, Hha, and Cnu are necessary for its conjugation repression activity. To this end, we performed filter paper conjugation using R6K/R6K∆*sfx-*carrying donors with *hns, stpA, hha,* or *cnu* deletions (gene deletions verified by lack of PCR amplification). We find that *hns, stpA,* and *cnu* deletions do not affect the conjugation efficiency of R6K and R6K∆*sfx* (Fig. 6D). In contrast, *hha* deletion increases the rate of R6K conjugation by ~10-fold, and this elevated conjugation efficiency is decreased by the complementation of Hha encoded on a low-copy vector (Fig. 6D). Moreover*, hha* deletion does not elevate the conjugation rate of R6K∆*sfx*, indicating that Hha repression of R6K conjugation is dependent on Sfx (Fig. 6D). This result and our bacterial two-hybrid findings indicate that Sfx may interact with Hha to repress R6K conjugation.

### Sfx loss does not affect R6K fitness in laboratory settings

Conjugation is an energetically expensive process that can sometimes decrease the fitness of plasmid carriers (96, 97). We have so far been unable to detect a fitness cost (i.e., growth defect in wild-type *E. coli*) associated with carrying R6K∆*sfx* in liquid culture (Fig. 4B), which is inconsistent with the expected tradeoff between conjugation and growth (98) and the widespread prevalence of Sfx homologs across MPF_T_-type IncX plasmids (Fig. S1). Therefore, we next investigated whether this lack of fitness cost of Sfx loss also applies in biofilms, a conjugative-permissive environment where plasmid fitness is dictated by donor (vertical inheritance) and transconjugant (horizontal inheritance) population dynamics. When co-cultured with EcoR25 recipient cells, we find that R6K and R6K∆*sfx* donors reached a similar population density in the biofilm and planktonic states (Fig. S4A). This result is affirmed by an R6K/R6K∆*sfx* competition assay, where both plasmid carriers reached a similar population density in the biofilm and planktonic states (Fig. S4B). Overall, we cannot detect a fitness cost to Sfx loss in a laboratory biofilm setting.

**Fig 7:**
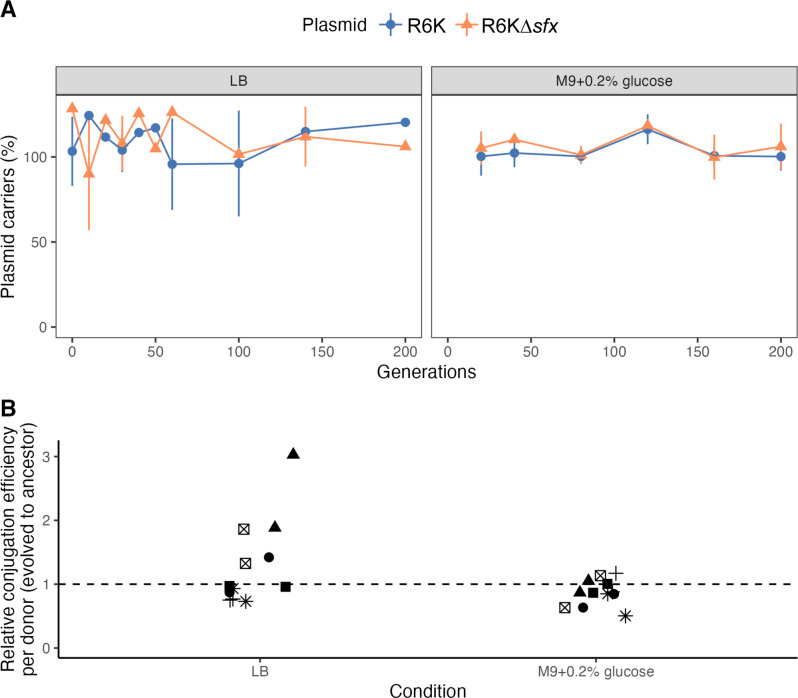
Sfx loss does not affect R6K stability. (**A**) R6K and R6K∆*sfx* are stably maintained throughout serial passaging in LB and M9 media. For each passaging experiment, six independent lineages are diluted 1:1,000 in fresh media without antibiotics every 24 h. The proportion of plasmid carriers is assessed by dividing the number of streptomycin-resistant colonies by the total colony count. Each point represents the mean proportion of plasmid carriers, and the lines delineate the mean ± standard deviation. (**B**) Passaging in LB or M9 does not decrease donor conjugation efficiency. The filter paper conjugation experiment is performed using the ancestral (day 0) and evolved (day 20) strains from the passaging experiment shown in panel **A**. The relative conjugation efficiency of the evolved-to-ancestor strain is plotted on the *y*-axis. The different shapes indicate the lineage of each sample, and each point represents the value of a biological replicate (averaged across two technical replicates).

We next investigated whether Sfx loss would incur a fitness cost on a longer timescale. To test this, we serially passaged six independent strains of *E. coli* BW25113 carrying R6K or R6K∆*sfx* in LB or minimal M9 + 0.2% glucose media for 20 days (~200 generations). Under both media conditions, all lineages stably maintained R6K and R6K∆*sfx* (Fig. 7A)*,* suggesting that Sfx loss does not impact plasmid stability within the study period, consistent with previous studies of an IncX3 plasmid (34). To investigate if compensatory mutations on the plasmids could account for this stability, we performed whole plasmid sequencing of the ancestor and two independent evolved lineages for each plasmid and passaging condition (a total of four evolved R6K and four R6K∆*sfx* sequenced). No mutations were detected on the plasmids after 20 days of serial passaging. To investigate whether the passaging selected for chromosomal mutations that affected plasmid conjugation efficiency, we selected two colonies (R6K∆*sfx* carriers) from each lineage and passaging condition and compared their conjugation rate relative with their ancestors. We find that passaging in LB and M9 media did not impact the conjugation efficiency of R6K∆*sfx* carriers (one lineage passaged in LB displayed slightly higher conjugation efficiency; Fig. 7B), suggesting that short-term passaging did not select for lower conjugation efficiency. This data is consistent with our inability to detect a fitness cost associated with Sfx loss.

## DISCUSSION

Plasmid conjugation is ubiquitous in natural bacterial communities and is crucial in driving bacterial evolution (1, 99–101). Interestingly, conjugation is repressed by default across most natural conjugative plasmids (7), indicating widespread selective pressures that drove the convergent evolution of repression systems. Understanding the molecular mechanism and evolution of conjugation repression is of major interest in plasmid biology. Here, we report that the evolutionary divergence of Sfx, a plasmid-encoded H-NS homolog, contributes to the unique regulation of IncX plasmid conjugation.

Gene duplication has long been recognized as a major driver for genome evolution (102, 103). Duplicated genes, known as paralogs, provide functional redundancy that temporarily relaxes purifying selection, allowing for sequence divergence that leads to gene pseudogenization (becoming non-functional), conservation (maintaining the same function), subfunctionalization (performing a subset of roles of the ancestral gene), or neofunctionalization (performing a new function) (103). Our results suggest that this duplication-divergence framework may explain the evolution of Sfx and the unique regulatory system of some IncX plasmids.

First, our phylogenetic analysis indicates that Sfx homologs form the sister clade of the H-NS paralog StpA (Fig. 1A), consistent with prior work (35). This relationship suggests that ancestral Sfx may also be a H-NS paralog and likely experienced reduced purifying selection following the duplication. Indeed, our molecular evolution analysis suggests that the extant StpA clade experiences more relaxed selective constraints relative to other H-NS homologs (Fig. S2), presumably due to the lack of a pronounced fitness cost associated with *stpA* null mutations (77, 104). We propose that this recessive phenotype of StpA, and likely other H-NS paralogs, potentiates the evolution of functionally distinct H-NS proteins.

Both neutral evolution (105) and positive selection (106–108) are known to drive sequence divergence following gene duplication. What drove the divergence of ancestral Sfx following its duplication? Our evolutionary analyses suggest that positive selection contributed to the initial burst of evolution experienced by the ancestral Sfx lineage (Fig. 2). However, neutral evolution dominated in the clade’s later evolution (Table S3), implying the loss of functional redundancy following lineage divergence. We propose that this later neutral evolution of the Sfx clade may be facilitated by the proliferation of homologs across IncX plasmids, particularly those with the MPF_T_-type T4SS, with plasmid incompatibility curbing gene recombination and a higher gene copy number potentially boosting mutation rates (109, 110).

Having established the mechanism of Sfx clade evolution, we next explored the functional consequences of these mutations. Indeed, many of the positively selected sites we document align with H-NS regions important for dimerization, oligomerization, interaction with Hha, DNA binding, and environmental sensing (Table 1). Some of these variations underpin the functional divergence between Sfx and chromosomal H-NS. Notably, Sfx, but not H-NS nor StpA, can repress conjugation of R6K (Fig. 3D), a feature that likely favored the migration/persistence of ancestral Sfx onto/on IncX plasmids. The C-terminal DNA-binding domain of Sfx is necessary for conjugation repression. Additionally, Sfx-mediated repression is enhanced by its interaction with chromosomal Hha and requires Sfx’s N-terminal dimerization domain (Fig. 6A). This co-repression may involve Hha’s ability to promote DNA bridging and generate topological stress (78, 111), inhibiting conjugation gene transcription and/or prohibiting the initial unwinding of the donor strand needed for conjugative transfer. Conversely, plasmid-encoded topoisomerase III (*topB*), which frequently colocalizes with *sfx* (36, 37)*,* could relieve this topological stress and promote conjugation. Given that similar Hha-H-NS co-repression is observed in the IncHI plasmid R27 (73) and that Hha homologs are often co-localized with H-NS homologs in plasmids (89), conjugation regulation by H-NS/Hha pairs may be a common mechanism.

After demonstrating the functional importance of Sfx’s C-terminal DNA-binding domain, we further explored the biochemical basis of its significance. Our gain-of-function experiments indicate that the linker region is important for Sfx’s conjugation repression activity (Fig. 5C). Positive selection within the linker region notably redistributed its positive charge (Fig. 2B). Although the functional impact of this charge variation is unclear—since similar changes in chromosomal H-NS did not significantly impact its regulatory activity (64)—it is plausible that the distinct charge distribution, longer length, and increased proline-induced rigidity (41) of the Sfx linker could collectively contribute to its unique repression activity.

The RGR AT-hook motif is another distinct feature of the Sfx clade (Fig. 1A) and contributes to the regulatory activity of Sfx’s C-terminal DNA binding domain (Fig. 5C). The first position of this motif displays signatures of positive selection (Fig. 2A). Remarkably, altering just one amino acid from QGR to RGR in chromosomal H-NS enables it to partially repress R6K conjugation (Fig. 5C), demonstrating that small sequence changes can confer large functional shifts. Interestingly, the RGR AT-hook motif is typically found in distantly related H-NS-like proteins encoded by bacteria with GC-rich genomes. These include Lsr2 carried by *Mycobacteria* (62-70% GC) (112) and Bv3F carried by *Burkholderia* (40) (59.2%–68.9% GC; from NCBI genome). These H-NS-like proteins show a heightened affinity for higher GC% sequences than those with the QGR motif (40, 66), enabling them to better differentiate between core and foreign genes in high GC% genomic contexts (66, 113).

Curiously, Sfx homologs are not found in high GC% genomic contexts (Fig. S5B). For instance, R6K has a GC% of 45.3%, slightly lower than *E. coli* BW25113 (50.8%; NCBI accession: CP009273.1). Furthermore, IncX plasmids carrying Sfx homologs display an unusual base composition: their insertion elements (foreign cargo genes) are higher in GC% than their core genes (median GC% difference of 7.4%; Fig. S5A and B), unlike previously characterized plasmids (114, 115). We hypothesize that Sfx’s RGR AT-hook motif and the plasmid’s atypical GC composition may facilitate better regulation of plasmid conjugation. First, the transfer operon of R6K is less AT-rich than typical H-NS binding sites (56.6% AT from *taxA* to *tiv11* vs. 61.4% AT for average H-NS-bound site [116]). Therefore, the RGR motif may enable Sfx to bind and repress transcription more effectively in these regions, where the higher GC content prohibits efficient regulation by chromosomal H-NS. This bias toward Sfx-mediated regulation, rather than H-NS-mediated, could confer several selective advantages, including optimized timing of conjugation independent of H-NS activity and adaptability to hosts without H-NS homologs. The elevated GC% of insertion elements also suggests potential decoupling of insertion element regulation from conjugation, enabling appropriate cargo gene expression that enhances plasmid carrier fitness. Overall, Sfx represents a unique instance of an RGR-bearing H-NS homolog found in a lower GC% genomic context. Future experiments examining the exact mechanism of how the RGR AT-hook motif facilitates conjugation regulation could yield invaluable insights.

Conjugation repression is common among characterized plasmids (7), and the fitness cost of constitutive conjugation is well-documented in numerous plasmid systems (9, 10, 96–98, 117). However, our results did not reveal any apparent fitness penalty associated with Sfx loss. *E. coli* carrying R6K or R6K∆*sfx* display similar growth dynamics in nutrient-rich (LB) and -poor (M9) environments (Fig. 4B through E; Fig. S4), and R6K∆*sfx* carriers exhibit growth defects only under highly artificial conditions in which chromosomal H-NS is also lost (Fig. 4D). This lack of fitness cost is also found on a larger time scale, wherein R6K∆*sfx* is stably maintained in the absence of antibiotic selection, consistent with previous studies on IncX3 plasmids (34), without selecting for mutations affecting conjugation efficiency (Fig. 7). Our findings suggest that conjugatively derepressed plasmids may have a fitness advantage under certain conditions. Indeed, plasmids harboring “superspreader” mutations that increase conjugation efficiency have been isolated from clinical and environmental sources (118, 119). Our findings also imply that other selective forces, not measured in laboratory conditions, may impose a selective advantage to keeping plasmid conjugation rates low, such as susceptibility to bacteriophages that attach to conjugal pili (12–15).

In summary, our study provides insights into the molecular evolution and functionality of plasmid-encoded H-NS homologs. We demonstrate the evolutionary and functional divergence of Sfx from chromosomal H-NS and its unique role in plasmid conjugation repression. The altered protein-protein and protein-DNA interaction interfaces of Sfx, driven partially by positive selection, likely contribute to its distinct functionality. Despite the conservation of Sfx among IncX plasmids, its loss does not impose an obvious fitness cost, reflecting the complexity of selection forces shaping plasmid evolution. Our results, therefore, highlight the sequence and functional diversity within the H-NS family and underscore the pivotal role that protein evolution plays in plasmid biology.

## MATERIALS AND METHODS

### Strains and plasmids

The strains, plasmids, and oligonucleotides used in this study are listed in Table S4 through S6, respectively. *E. coli* DH5α (NEB) was used for cloning and plasmid propagation. Except for the bacterial two-hybrid assays, all experiments were performed using the *E. coli* BW25113 or strains from the Keio collection when specified (120). For all conjugation assays, the recipient strain was a spontaneous NaN_3_^r^ EcoR25 strain derived in-house. *E. coli* BTH101 (F−, *cya-99, araD139, galE15, galK16, rpsL1* [Str^r^], *hsdR2, mcrA1, mcrB1*) and T18/T25-fragment containing plasmid backbones (pKT25, pKNT25, pUT18, pUT18C) are a kind gift from Véronique Taylor (University of Toronto) and were used for the bacterial two-hybrid assays. The domain-swapping, complementation, and bacterial two-hybrid plasmids were constructed via Gibson assembly using PCR-amplified vector backbone and fragments from R6K or *E. coli* BW25113 genomic DNA following the manufacturer’s instructions. Mutations and small fragment substitutions in plasmids were introduced via site-directed mutagenesis using a homebrew KLD enzyme mix. Plasmids were transformed into *E. coli* using electroporation or heat shock. R6K∆*sfx* is a kind gift from Irina Artsimovitch (Ohio State University). All plasmid constructs were verified via Sanger or Nanopore sequencing (Plasmidsaurus).

### Media and culture conditions

*E. coli* strains were routinely grown in LB liquid media (BD Difco LB Broth Miller) or LB agar (BD Difco LB Agar Miller) at 37˚C. Liquid cultures were grown with shaking (200 rpm). When required for plasmid maintenance, the growth medium was supplemented with 100 µg/mL streptomycin, 50 µg/mL kanamycin, 100 µg/mL ampicillin, 20 µg/mL chloramphenicol, and/or 0.01% NaN_3_. For growth curve, biofilm, and serial passaging, strains were grown in M9 (BD Difco Bacto M9 Minimal Salts) + 0.2% glucose when specified.

### Constructing phylogeny of Sfx, H-NS, and StpA

A Sfx, H-NS, and StpA phylogeny was inferred via the maximum-likelihood method using IQ-TREE2 (121). The best codon substitution model was chosen using ModelFinder (122), and branch confidence was assessed via 5,000 rounds of Ultrafast Bootstrap Approximation (123) and 1,000 bootstrap replicates of SH-aLRT (124). For visualization purposes, the tree was rooted at the Ler protein, but all molecular evolution analyses were performed on an unrooted phylogeny that does not contain the Ler protein sequence. Sequence taxonomy was derived based on the subfamily classification proposed by Alnajar and Gupta (125), whereas other sequence characteristics (genetic context, AT-hook motif) were retrieved from NCBI or derived from the protein alignment and mapped onto the phylogeny using the ggtree package (126).

### Conservation and alignment of chromosomal and plasmid-borne H-NS homologs

We aligned the amino acid sequences of Sfx (NCBI accession ID: WP_001282381.1), Acr2 (NCBI accession ID: WP_000651490.1), StpA (Uniprot accession: P0ACG1), Sfh (Uniprot accession: Q8GKU0), and H-NS (Uniprot accession: P0ACF8) using MAFFT G-INS-i (127). The conservation within Sfx homologs was derived using the ConSurf webserver (128) with default parameters.

### Mapping Sfx orthologs across IncX plasmids

We retrieved 975 IncX plasmids, as classified by PlasmidFinder (129), from PLSDB (130) in March of 2023. One plasmid was arbitrarily selected from each unique PlasmidFinder lineage to produce 55 representative sequences. For each lineage representative, we reannotated the sequences using PGAP (131) and derived plasmid attributes (replicon, relaxase, MPF, OriT, predicted mobility, predicted host range) using MOB-suite (132). To identify H-NS orthologs and co-occurring proteins, we performed orthologous clustering of all 55 lineages using OrthoFinder with default parameters (133). The AT-hook motif of the H-NS homologs was derived by performing multiple sequence alignment using the DECIPHER package (134) and further validated by visual inspection. The attributes of the representative plasmids were clustered via hierarchical clustering using Gower distance and visualized using the R package ggtree (126).

### Generating Sfx, H-NS, and StpA sequence alignment for molecular evolution analysis

The NCBI nr/nt database (135) is queried using tblastn in October 2022 with default search parameters to retrieve nucleotide sequence homologous to Sfx from the R6K plasmid (NCBI accession ID: WP_001282381.1), H-NS from *E. coli* K12 (Uniprot ID: P0ACF8), and StpA from *E. coli* K12 (Uniprot ID: P0ACG1). We removed all hits that are <50% similar to the seed template sequences and retrieved the coding sequences corresponding to the remaining hits using an in-house script. The resulting sequences were filtered to eliminate short (<88 amino acids for Sfx homologs, <100 amino acids for H-NS and StpA homologs) and overly long peptides (>200 amino acids for all sequences) that are indicative of pseudogenes and/or misannotation. The nucleotide sequences were clustered using MMseqs2 (136) to identify sequence-level representatives (95% identity, 80% coverage, coverage mode = 1). The sequence representatives were aligned using the Guidance2 web server (137) (codon model, MAFFT with maximum 1,000 cycles of iterations) and trimmed to eliminate any sequences with <0.6 confidence score and columns with <0.87 confidence score. To aid with phylogeny visualization, the Ler protein from *E. coli* O157:H7 str. Sakai (GenBank accession: BAB38011.2), a H-NS-like protein that is distantly related to Sfx, H-NS, and StpA (138) (pairwise sequence identity ~20%–25%) was chosen as the outgroup and aligned with all sequences.

### Molecular evolution analysis using PAML

We used PAML 4.10.6 (139) to explore the variation in selective constraints across Sfx, H-NS, and StpA homologs. Branch-sites (140) and clade models (141) were fitted using the codeml program, and likelihood ratio tests were used to explore whether using more complex models leads to a statistically significant improvement in model fit (142). We used the Akaike information criterion (AIC) as an evaluation metric for comparison across non-nested models, as suggested by Weadick and Chang (142). Residues under positive selection were detected using NEB and BEB analyses (46).

### AlphaFold structural prediction of Sfx, H-NS, and StpA

Tetramers and dimers of Sfx, H-NS, and StpA were predicted using ColabFold (Alphafold2-multimer) (143). Template information of each sequence set was fetched from the PDB70 database, and the predicted structures were relaxed using the amber force fields. The secondary structure was derived using POLYVIEW-2D (144) and visualized using the R package gggenes (145), whereas the 3D structure was modeled in PyMOL (146).

### Constructing WebLogo of Sfx, H-NS, and StpA homologs

Sequences within the Sfx, H-NS, and StpA clades (defined based on phylogeny) were aligned (within-clade) using the MAFFT E-INS-i algorithm (127). The sequence alignments were manually trimmed using Pfaat to remove gappy regions (147), and the amino acid frequency at each position was visualized using the WebLogo webserver (148).

### Filter paper conjugation assay

For all experiments, one biological replicate of the recipient strain and two biological replicates of the donor strains were grown overnight in LB (recipient: supplemented with 0.01% NaN_3_; donor: supplemented with 100 µg/mL of streptomycin, and 20 µg/mL chloramphenicol if harboring pHSG576 constructs) at 37˚C with shaking (200 rpm). The conjugation efficiency on filter paper was assessed as follows: 2 OD_600_ U of donor and recipient (spontaneous NaN_3_^r^ EcoR25) overnight cultures were pelleted (4,500 × *g* for 5 min at room temperature), washed with sterile phosphate-buffered saline (PBS), and combined at equal volume. In addition, 20 µL of the culture mixture was spotted on MF-Millipore Membrane Filter (0.22 µm pore size) overlaid on LB agar and incubated at 30°C for 3 h. The bacterial spots were washed off by vortexing the filter paper discs in 100 µL of sterile PBS for 20 s. The donor and transconjugant populations were determined by spotting serially diluted bacterial mixtures on LB agar supplemented with 100 µg/mL ampicillin and LB agar supplemented with 100 µg/mL ampicillin and 0.01% NaN_3_, respectively. Each conjugation experiment was repeated at least twice.

### Biofilm conjugation assay

Donor and recipient strains were grown and processed via the same method outlined in “Filter paper conjugation assay.” For each strain, washed overnights of three biological replicates were diluted to a final OD_600_ of 0.03 in 150 µL of M9 + 0.2% glucose media in a 96-well polystyrene plate (Sarstedt). The similarity in donor cell input (for conjugation assays) or R6K/R6K∆*sfx* carrier (for biofilm competition assay) was validated by plating serially diluted inoculum on LB agar supplemented with 100 µg/mL streptomycin (which selects for both R6K and R6K∆*sfx* carriers) and/or 50 µg/mL kanamycin (which selects for only R6K∆*sfx* carriers). Biofilm formation was allowed to occur statically at 30˚C for 24 h. After 24 h, the planktonic cultures were aspirated and quantified via selective plating (see below for conditions). The biofilm was gently washed once with 150 µL of sterile PBS to remove non-adherent cells and resuspended in 40 µL of sterile PBS. To assess donor, recipient, and transconjugant population, the planktonic cultures/resuspended biofilms were serial diluted and spotted on LB agar supplemented with 100 µg/mL ampicillin, or 0.01% NaN_3_, or 100 µg/mL ampicillin and 0.01% NaN_3_, respectively. Each biofilm conjugation experiment was repeated at least twice.

### Motility assay

Overnight *E. coli* cultures grown at 37˚C with shaking were subcultured 1:100× in 5 mL of LB with no antibiotics to an OD_600_ of ~0.6. Six microliters of mid-log culture was aspirated into the middle of 0.3% LB agar, dried at room temperature for 1 h, and incubated at 30˚C for 24 h. The motility assay was repeated twice.

### Growth curve analysis

One OD_600_ unit of overnight *E. coli* cultures were pelleted (4,500 × *g* for 5 min at room temperature), washed with sterile PBS, and diluted to a final OD_600_ of 0.02 in 200 µL of growth media (LB or M9 + 0.2% glucose supplemented with 20 µg/ml chloramphenicol if strains carry pHSG576 constructs). All cells were seeded in 96-well polystyrene plates (Sarstedt), sealed with Breathe-Easy sealing membrane (Sigma Aldrich), and grown at 37˚C for 24 h with shaking every 15 min. OD_620_ was monitored every 15 min by an S&P growth robot. All growth curve assays were repeated at least three times.

### Bacterial two-hybrid assay

The coding sequences of *hns, stpA*, *hha*, and *cnu* were amplified via PCR from *E. coli* BW25113 genomic DNA, whereas the coding sequences of *sfx* was amplified from R6K. The PCR products were cloned into bacterial two-hybrid vectors (pUT18, pUT18C, pKT25, pKNT25) using Gibson assembly following the manufacturer’s instructions and validated by Sanger sequencing. All H-NS homolog constructs (Sfx, H-NS, and StpA) were fused to the adenylate cyclase fragment at its C-terminus to preserve the N-terminal dimerization domain. High-copy vectors (pUT18C) encoding for Hha and Cnu are constructed with only the N-terminal T18 fusion (given that C-terminal fusion disrupts H-NS-Hha interaction [94]), whereas both N-terminal and C-terminal chimeric proteins were constructed for low-copy vectors. To detect protein-protein interactions, we heat-shocked the bacterial two-hybrid vectors into *E. coli* BTH101 and plated them on LB supplemented with 100 µg/mL ampicillin and 50 µg/mL kanamycin to select for transformants. Three biological replicates of each strain were grown overnight in LB supplemented with 100 µg/mL ampicillin and 50 µg/mL kanamycin at 37°C with shaking (200 rpm). Expression of the chimeric proteins was then induced by spotting 5 µL of overnight cultures on LB agar supplemented with 100 µg/mL ampicillin, 50 µg/mL kanamycin, 40 µg/mL X-gal, and 50 mM isopropyl β-D-1-thiogalactopyranoside (IPTG). The plates are incubated at 30°C for 24 h (for strains carrying pUT18::*hns* (M1-G85), pUT18::*stpA,* pUT18::*sfx,* pUT18::*sfx-*N-truncated) or 48 h (for strains carrying pUT18C::*hha* and pUT18C::*cnu*). Each bacterial two-hybrid screen was repeated twice.

### Serial passaging and assessing passaged strain conjugation efficiency

Six independent lineages of *E. coli* carrying R6K or R6K∆*sfx* were serially passaged in LB or M9 + 0.2% glucose for 20 days (~200 generations). All strains were grown in 5 mL of media in 20 mm glass tubes at 37˚C with shaking (200 rpm). Every 24 h, the cultures were diluted 1:1,000× in fresh media. The proportion of plasmid-retaining cells was assessed by plating serially diluted cultures on LB agar (quantify total bacterial population) and LB agar supplemented with 100 µg/mL streptomycin (which selects for only plasmid carriers). To assess whether mutations accumulated on the R6K/R6K∆*sfx* plasmid during passaging, single colonies of passaged strain (from frozen DMSO stocks) were grown overnight in LB supplemented with 100 µg/mL streptomycin. Plasmids were harvested using the GeneJET Plasmid Miniprep Kit following the manufacturer’s protocol and sequenced using Nanopore sequencing (Plasmidsaurus). The effect of serial passaging on plasmid conjugation was assessed by taking single colonies from day 0 (ancestral strain) and day 20 (passaged strain) DMSO stocks preserved at −80˚C and evaluating the filter paper conjugation efficiency of two biological replicates, each with two technical replicates (filter paper conjugation experiment was performed as specified above).

### GC content calculation of IS and non-IS regions in IncX plasmids

We used the ISfinder algorithm (blastn, default parameters) (149) to search for putative IS regions across 22 IncX plasmid representatives with H-NS homologs. The resulting hits were filtered to retain only higher confidence (e-value ≤ 0.1) and longer sequences (>100 bp). An in-house script was then used to calculate the GC% of all putative IS and non-IS regions.

## SUPPLEMENTAL MATERIAL

Supplemental Tables mbio.02089-24-s0001.docx Tables S1 to S6.

Supplemental Figures mbio.02089-24-s0002.docx Figures S1 to S5.

ASM does not own the copyrights to Supplemental Material that may be linked to, or accessed through, an article. The authors have granted ASM a non-exclusive, world-wide license to publish the Supplemental Material files. Please contact the corresponding author directly for reuse.
