# Supplementary material for: Evolutionary and functional divergence of Sfx, a plasmid-encoded H-NS homolog, underlies the regulation of IncX plasmid conjugation
Source: mBio. 2024 Dec 23;16(2):e02089-24. doi: 10.1128/mbio.02089-24 (PMC11796372; doi:10.1128/mbio.02089-24)
Supplement: Supplemental Tables — Tables S1 to S6. [file mbio.02089-24-s0001.docx]

**Supplementary Tables**

**Table S1. Characteristics of Sfx, H-NS, and StpA representative homologs.**

| **ID** | **Scaffold accession** | **Seed** | **Family** | **Species** | **Genomic context** | **Motif** | **Clade** |
| --- | --- | --- | --- | --- | --- | --- | --- |
| hnsx_01 | AP008233.1 | Sfx | *Bruguierivoracaceae* | *Sodalis glossinidius str. 'morsitans'* | Plasmid | KGR | Sfx |
| hnsx_02 | AP019678.1 | Sfx | *Enterobacteriaceae* | *Escherichia coli* | Plasmid | RGR | Sfx |
| hnsx_03 | AP021965.1 | Sfx | *Enterobacteriaceae* | *Escherichia coli* | Plasmid | RGR | Sfx |
| hnsx_04 | AP022496.1 | Sfx | *Enterobacteriaceae* | *Citrobacter portucalensis* | Plasmid | RGR | Sfx |
| hnsx_06 | CP008799.1 | Sfx | *Enterobacteriaceae* | *Klebsiella pneumoniae subsp. pneumoniae KPNIH24* | Plasmid | RGR | Sfx |
| hnsx_07 | CP010193.1 | Sfx | *Enterobacteriaceae* | *Escherichia coli* | Plasmid | RGR | Sfx |
| hnsx_08 | CP010344.1 | Sfx | *Enterobacteriaceae* | *Escherichia coli ECC-1470* | Chromosome | QGR | H-NS |
| hnsx_09 | CP010344.1 | Sfx | *Enterobacteriaceae* | *Escherichia coli ECC-1470* | Chromosome | QGR | H-NS |
| hnsx_10 | CP013970.1 | Sfx | *Erwiniaceae* | *Erwinia tracheiphila* | Chromosome | RGR | Sfx |
| hnsx_11 | CP017584.1 | Sfx | *Erwiniaceae* | *Pantoea stewartii subsp. stewartii DC283* | Plasmid | RGR | Sfx |
| hnsx_12 | CP019905.1 | Sfx | *Enterobacteriaceae* | *Escherichia coli* | Plasmid | NA | Sfx |
| hnsx_13 | CP023675.1 | Sfx | *Enterobacteriaceae* | *Escherichia coli* | Plasmid | RGR | Sfx |
| hnsx_14 | CP025255.1 | Sfx | *Enterobacteriaceae* | *Salmonella enterica subsp. enterica serovar Newport str. CDC 2010K-2159* | Plasmid | RGR | Sfx |
| hnsx_15 | CP028735.1 | Sfx | *Enterobacteriaceae* | *Escherichia coli* | Chromosome | RGR | Sfx |
| hnsx_16 | CP030195.1 | Sfx | *Enterobacteriaceae* | *Salmonella enterica* | Plasmid | RGR | Sfx |
| hnsx_17 | CP032087.1 | Sfx | *Enterobacteriaceae* | *Escherichia coli* | Plasmid | RGR | Sfx |
| hnsx_18 | CP042976.1 | Sfx | *Enterobacteriaceae* | *Klebsiella pneumoniae* | Plasmid | KGR | Sfx |
| hnsx_19 | CP045055.1 | Sfx | *Enterobacteriaceae* | *Salmonella enterica subsp. enterica serovar Muenchen* | Plasmid | RGR | Sfx |
| hnsx_20 | CP049602.1 | Sfx | *Enterobacteriaceae* | *Klebsiella aerogenes* | Plasmid | RGR | Sfx |
| hnsx_21 | CP056187.1 | Sfx | *Enterobacteriaceae* | *Citrobacter sp. RHBSTW-01013* | Plasmid | RGR | Sfx |
| hnsx_22 | CP056812.1 | Sfx | *Enterobacteriaceae* | *Escherichia coli* | Plasmid | RGR | Sfx |
| hnsx_23 | CP063689.1 | Sfx | *Erwiniaceae* | *Erwinia amylovora* | Plasmid | RGR | Sfx |
| hnsx_24 | CP070064.1 | Sfx | *Enterobacteriaceae* | *Escherichia coli* | Plasmid | RGR | Sfx |
| hnsx_25 | CP070509.1 | Sfx | *Yersiniaceae* | *Serratia ureilytica* | Plasmid | VGR | Sfx |
| hnsx_26 | CP088523.1 | Sfx | *Enterobacteriaceae* | *Escherichia coli* | Plasmid | RGR | Sfx |
| hnsx_27 | CP092255.1 | Sfx | *Enterobacteriaceae* | *Escherichia coli* | Plasmid | RGR | Sfx |
| hnsx_28 | FJ914220.1 | Sfx | *Enterobacteriaceae* | *Escherichia coli* | Plasmid | RGR | Sfx |
| hnsx_29 | KU963390.1 | Sfx | *Enterobacteriaceae* | *Escherichia coli* | Plasmid | RGR | Sfx |
| hnsx_30 | KX683284.1 | Sfx | *Enterobacteriaceae* | *Escherichia coli* | Plasmid | NA | Sfx |
| hnsx_31 | MT039145.1 | Sfx | *Yersiniaceae* | *Serratia entomophila* | Plasmid | VGR | Sfx |
| hnsx_32 | MZ513635.1 | Sfx | *Enterobacteriaceae* | *Escherichia coli* | Plasmid | RGR | Sfx |
| hnsx_33 | OW995938.1 | Sfx | *Enterobacteriaceae* | *Escherichia coli* | Plasmid | RGR | Sfx |
| hns_01 | CP048860.1 | H-NS | *Enterobacteriaceae* | *Escherichia coli* | Chromosome | QGR | H-NS |
| hns_02 | AP026087.1 | H-NS | *Enterobacteriaceae* | *Escherichia coli* | Chromosome | QGR | H-NS |
| hns_03 | CP055675.1 | H-NS | *Enterobacteriaceae* | *Escherichia fergusonii* | Chromosome | QGR | H-NS |
| hns_04 | CP093076.1 | H-NS | *Enterobacteriaceae* | *Salmonella enterica* | Chromosome | QGR | StpA |
| hns_05 | AP024281.1 | H-NS | *Enterobacteriaceae* | *Enterobacter asburiae* | Chromosome | QGR | H-NS |
| hns_06 | CP083643.1 | H-NS | *Enterobacteriaceae* | *Enterobacter bugandensis* | Chromosome | QGR | H-NS |
| hns_07 | CP090133.1 | H-NS | *Enterobacteriaceae* | *Salmonella enterica subsp. enterica serovar Goldcoast* | Chromosome | QGR | H-NS |
| hns_08 | CP056896.1 | H-NS | *Enterobacteriaceae* | *Citrobacter sp. RHBSTW-00021* | Chromosome | QGR | StpA |
| hns_09 | CP024673.1 | H-NS | *Enterobacteriaceae* | *Citrobacter freundii* | Chromosome | QGR | H-NS |
| hns_10 | CP053045.1 | H-NS | *Enterobacteriaceae* | *Escherichia fergusonii* | Chromosome | QGR | StpA |
| hns_11 | CP007298.2 | H-NS | *Enterobacteriaceae* | *Salmonella enterica subsp. enterica serovar Enteritidis str. EC20090530* | Chromosome | QGR | H-NS |
| hns_12 | CP056341.1 | H-NS | *Enterobacteriaceae* | *Citrobacter sp. RHBSTW-00696* | Chromosome | QGR | H-NS |
| hns_13 | CP056168.1 | H-NS | *Enterobacteriaceae* | *Enterobacter roggenkampii* | Chromosome | QGR | StpA |
| hns_14 | CP070296.2 | H-NS | *Enterobacteriaceae* | *Escherichia albertii* | Chromosome | QGR | StpA |
| hns_15 | CP014070.2 | H-NS | *Enterobacteriaceae* | *Citrobacter amalonaticus* | Chromosome | QGR | StpA |
| hns_16 | OW849320.1 | H-NS | *Enterobacteriaceae* | *Enterobacter cloacae* | Chromosome | QGR | StpA |
| hns_17 | CP033780.1 | H-NS | *Enterobacteriaceae* | *Citrobacter koseri* | Chromosome | QGR | H-NS |
| hns_18 | CP013190.1 | H-NS | *Enterobacteriaceae* | *Escherichia coli* | Chromosome | QGR | H-NS |
| hns_19 | CP039453.1 | H-NS | *Enterobacteriaceae* | *Enterobacter bugandensis* | Chromosome | QGR | StpA |
| hns_20 | CP044101.1 | H-NS | *Enterobacteriaceae* | *Citrobacter werkmanii* | Chromosome | QGR | StpA |
| hns_21 | CP056199.1 | H-NS | *Enterobacteriaceae* | *Enterobacter sp. RHBSTW-00994* | Chromosome | QGR | StpA |
| hns_22 | AP022133.1 | H-NS | *Enterobacteriaceae* | *Enterobacter cloacae* | Chromosome | QGR | StpA |
| hns_23 | CP026709.1 | H-NS | *Enterobacteriaceae* | *Citrobacter koseri* | Chromosome | QGR | StpA |
| hns_24 | CP088802.1 | H-NS | *Enterobacteriaceae* | *Escherichia coli* | Chromosome | QGR | H-NS |
| hns_25 | CP099898.1 | H-NS | *Enterobacteriaceae* | *Escherichia albertii* | Chromosome | QGR | H-NS |
| hns_26 | CP050811.1 | H-NS | *Enterobacteriaceae* | *Yokenella regensburgei* | Chromosome | QGR | H-NS |
| hns_27 | CP057688.1 | H-NS | *Enterobacteriaceae* | *Escherichia coli* | Chromosome | QGR | StpA |
| hns_28 | CP050811.1 | H-NS | *Enterobacteriaceae* | *Yokenella regensburgei* | Chromosome | QGR | StpA |
| hns_29 | CP059481.1 | H-NS | *Enterobacteriaceae* | *Enterobacter kobei* | Chromosome | QGR | StpA |
| hns_30 | CP064363.1 | H-NS | *Enterobacteriaceae* | *Salmonella enterica subsp. arizonae* | Chromosome | QGR | StpA |
| hns_31 | CP011132.1 | H-NS | *Enterobacteriaceae* | *Citrobacter amalonaticus Y19* | Chromosome | QGR | StpA |
| hns_32 | CP014015.2 | H-NS | *Enterobacteriaceae* | *Citrobacter amalonaticus* | Chromosome | QGR | H-NS |
| hns_33 | CP081314.1 | H-NS | *Enterobacteriaceae* | *Citrobacter farmeri* | Chromosome | QGR | StpA |
| hns_34 | CP058209.1 | H-NS | *Enterobacteriaceae* | *Escherichia marmotae* | Chromosome | QGR | StpA |
| hns_35 | AP025653.1 | H-NS | *Enterobacteriaceae* | *Citrobacter koseri* | Chromosome | QGR | H-NS |
| hns_36 | AP022256.1 | H-NS | *Enterobacteriaceae* | *Klebsiella sp. WP8-S18-ESBL-06* | Chromosome | QGR | H-NS |
| hns_37 | CP057808.1 | H-NS | *Enterobacteriaceae* | *Escherichia coli* | Chromosome | QGR | H-NS |
| hns_38 | CP006580.1 | H-NS | *Enterobacteriaceae* | *Enterobacter ludwigii* | Chromosome | QGR | StpA |
| hns_39 | CP027986.1 | H-NS | *Enterobacteriaceae* | *Enterobacter sichuanensis* | Chromosome | QGR | StpA |
| hns_40 | CP092635.1 | H-NS | *Enterobacteriaceae* | *Enterobacter cloacae* | Chromosome | QGR | H-NS |
| hns_41 | CP009756.1 | H-NS | *Enterobacteriaceae* | *Enterobacter cloacae* | Chromosome | QGR | StpA |
| hns_42 | CP074201.1 | H-NS | *Enterobacteriaceae* | *Salmonella enterica subsp. enterica serovar Eastbourne str. CFSAN001084* | Chromosome | QGR | H-NS |
| hns_43 | CP102500.1 | H-NS | *Enterobacteriaceae* | *Citrobacter youngae* | Chromosome | QGR | StpA |
| hns_44 | CP087880.1 | H-NS | *Enterobacteriaceae* | *Pseudocitrobacter corydidari* | Chromosome | QGR | H-NS |
| hns_45 | CP098325.1 | H-NS | *Enterobacteriaceae* | *Leclercia adecarboxylata* | Chromosome | QGR | StpA |
| hns_46 | AP026392.1 | H-NS | *Enterobacteriaceae* | *Klebsiella quasipneumoniae subsp. quasipneumoniae* | Chromosome | QGR | H-NS |
| hns_47 | LR134485.1 | H-NS | *Enterobacteriaceae* | *Citrobacter youngae* | Chromosome | QGR | H-NS |
| hns_48 | CP083824.1 | H-NS | *Enterobacteriaceae* | *Enterobacter ludwigii* | Chromosome | QGR | H-NS |
| hns_49 | CP098325.1 | H-NS | *Enterobacteriaceae* | *Leclercia adecarboxylata* | Chromosome | QGR | H-NS |
| hns_50 | CP087618.1 | H-NS | *Enterobacteriaceae* | *Klebsiella pneumoniae subsp. pneumoniae* | Chromosome | QGR | H-NS |
| hns_51 | AP023447.1 | H-NS | *Enterobacteriaceae* | *Enterobacter roggenkampii* | Chromosome | QGR | H-NS |
| hns_52 | CP018628.1 | H-NS | *Enterobacteriaceae* | *Lelliottia jeotgali* | Chromosome | QGR | H-NS |
| hns_53 | CP063441.1 | H-NS | *Enterobacteriaceae* | *Enterobacter asburiae* | Chromosome | QGR | H-NS |
| hns_54 | CP071070.1 | H-NS | *Enterobacteriaceae* | *Citrobacter sedlakii* | Chromosome | QGR | StpA |
| hns_55 | CP057125.1 | H-NS | *Enterobacteriaceae* | *Citrobacter sp. RHB36-C18* | Chromosome | QGR | H-NS |
| hns_56 | CP104014.1 | H-NS | *Enterobacteriaceae* | *Enterobacter sp. CP102* | Chromosome | QGR | StpA |
| hns_57 | AB915983.1 | H-NS | *Enterobacteriaceae* | *Escherichia coli O63:H6* | Chromosome | QGR | H-NS |
| hns_58 | CP083650.1 | H-NS | *Enterobacteriaceae* | *Citrobacter europaeus* | Chromosome | QGR | StpA |
| hns_59 | CP043318.1 | H-NS | *Enterobacteriaceae* | *Enterobacter chengduensis* | Chromosome | QGR | StpA |
| hns_60 | CP026975.1 | H-NS | *Enterobacteriaceae* | *Enterobacter cloacae complex sp.* | Chromosome | QGR | H-NS |
| hns_61 | CP014993.1 | H-NS | *Enterobacteriaceae* | *Enterobacter asburiae* | Chromosome | QGR | H-NS |
| hns_62 | LR134214.1 | H-NS | *Enterobacteriaceae* | *Citrobacter portucalensis* | Chromosome | QGR | StpA |
| hns_63 | CP083857.1 | H-NS | *Enterobacteriaceae* | *Enterobacter kobei* | Chromosome | QGR | H-NS |
| hns_64 | CP085642.1 | H-NS | *Enterobacteriaceae* | *Citrobacter freundii* | Chromosome | QGR | H-NS |
| hns_65 | CP056235.1 | H-NS | *Enterobacteriaceae* | *Citrobacter freundii* | Chromosome | QGR | H-NS |
| hns_66 | CP055995.1 | H-NS | *Enterobacteriaceae* | *Enterobacter roggenkampii* | Chromosome | QGR | H-NS |
| hns_67 | CP087618.1 | H-NS | *Enterobacteriaceae* | *Klebsiella pneumoniae subsp. pneumoniae* | Chromosome | QGR | StpA |
| hns_68 | CP026235.1 | H-NS | *Enterobacteriaceae* | *Citrobacter freundii complex sp. CFNIH3* | Chromosome | QGR | H-NS |
| hns_69 | CP082841.1 | H-NS | *Enterobacteriaceae* | *Kluyvera sp. CRP* | Chromosome | QGR | H-NS |
| hns_70 | CP050320.1 | H-NS | *Enterobacteriaceae* | *Enterobacter sp. SES19* | Chromosome | QGR | StpA |
| hns_71 | CP025225.1 | H-NS | *Enterobacteriaceae* | *Enterobacter cancerogenus* | Chromosome | QGR | H-NS |
| hns_72 | CP057483.1 | H-NS | *Enterobacteriaceae* | *Citrobacter sp. RHB25-C09* | Chromosome | QGR | H-NS |
| hns_73 | CP012871.1 | H-NS | *Enterobacteriaceae* | *[Enterobacter] lignolyticus* | Chromosome | QGR | H-NS |
| hns_74 | FR877557.1 | H-NS | *Enterobacteriaceae* | *Salmonella bongori NCTC 12419* | Chromosome | QGR | StpA |
| hns_75 | LR134138.1 | H-NS | *Enterobacteriaceae* | *Kluyvera intermedia* | Chromosome | QGR | H-NS |
| hns_76 | CP025699.1 | H-NS | *Enterobacteriaceae* | *Lelliottia sp. AC1* | Chromosome | QGR | H-NS |
| hns_77 | CP046445.1 | H-NS | *Enterobacteriaceae* | *Leclercia sp. 119287* | Chromosome | QGR | H-NS |
| hns_78 | CP087880.1 | H-NS | *Enterobacteriaceae* | *Pseudocitrobacter corydidari* | Chromosome | QGR | StpA |
| hns_79 | CP057611.1 | H-NS | *Enterobacteriaceae* | *Citrobacter sp. RHB21-C05* | Chromosome | QGR | H-NS |
| hns_80 | CP019245.1 | H-NS | *Enterobacteriaceae* | *Escherichia coli* | Chromosome | QGR | H-NS |
| hns_81 | CP102246.1 | H-NS | *Enterobacteriaceae* | *Enterobacter cloacae complex sp. R_G8* | Chromosome | QGR | StpA |
| hns_82 | CP104014.1 | H-NS | *Enterobacteriaceae* | *Enterobacter sp. CP102* | Chromosome | QGR | H-NS |
| hns_83 | CP082280.1 | H-NS | *Enterobacteriaceae* | *Enterobacter cancerogenus* | Chromosome | QGR | StpA |
| hns_84 | CP074170.1 | H-NS | *Enterobacteriaceae* | *Enterobacter sp. JBIWA003* | Chromosome | QGR | StpA |
| hns_85 | CP056474.1 | H-NS | *Enterobacteriaceae* | *Enterobacter cloacae* | Chromosome | QGR | H-NS |
| hns_86 | CP083824.1 | H-NS | *Enterobacteriaceae* | *Enterobacter ludwigii* | Chromosome | QGR | StpA |
| stpa_02 | CP062838.1 | StpA | *Enterobacteriaceae* | *Escherichia coli* | Chromosome | QGR | H-NS |
| stpa_03 | CP031215.1 | StpA | *Enterobacteriaceae* | *Escherichia coli* | Chromosome | QGR | H-NS |
| stpa_04 | CP041184.1 | StpA | *Enterobacteriaceae* | *Salmonella enterica subsp. enterica serovar Anatum* | Chromosome | QGR | H-NS |
| stpa_05 | CP079837.1 | StpA | *Enterobacteriaceae* | *Salmonella enterica subsp. salamae* | Chromosome | QGR | H-NS |
| stpa_06 | CP041302.1 | StpA | *Enterobacteriaceae* | *Escherichia coli* | Chromosome | QGR | H-NS |
| stpa_07 | CP073946.1 | StpA | *Enterobacteriaceae* | *Escherichia coli* | Chromosome | QGR | H-NS |
| stpa_08 | CP090277.1 | StpA | *Enterobacteriaceae* | *Escherichia coli* | Chromosome | QGR | H-NS |
| stpa_09 | CP004056.1 | StpA | *Enterobacteriaceae* | *Shigella flexneri 2003036* | Chromosome | QGR | H-NS |
| stpa_10 | CP082833.1 | StpA | *Enterobacteriaceae* | Citrobacter rodentium NBRC 105723 = DSM 16636 | Chromosome | QGR | StpA |
| stpa_11 | CP025757.1 | StpA | *Enterobacteriaceae* | *Citrobacter freundii complex sp. CFNIH2* | Chromosome | QGR | H-NS |
| stpa_12 | LR883000.1 | StpA | *Enterobacteriaceae* | *Escherichia coli* | Chromosome | QGR | H-NS |
| stpa_13 | CP019245.1 | StpA | *Enterobacteriaceae* | *Escherichia coli* | Chromosome | QGR | H-NS |
| stpa_14 | CP043487.1 | StpA | *Enterobacteriaceae* | *Escherichia coli* | Chromosome | QGR | StpA |
| stpa_15 | CP034745.1 | StpA | *Enterobacteriaceae* | *Escherichia coli* | Chromosome | QGR | H-NS |
| stpa_16 | CP080253.1 | StpA | *Enterobacteriaceae* | *Escherichia coli* | Chromosome | QGR | H-NS |
| stpa_17 | CP029989.1 | StpA | *Enterobacteriaceae* | *Salmonella enterica subsp. diarizonae serovar 48:i:z* | Chromosome | QGR | H-NS |
| stpa_18 | CP069159.1 | StpA | *Enterobacteriaceae* | *Citrobacter sp. R56* | Chromosome | QGR | StpA |
| stpa_19 | CP057166.1 | StpA | *Enterobacteriaceae* | *Escherichia coli* | Chromosome | QGR | H-NS |
| stpa_20 | CP063046.1 | StpA | *Enterobacteriaceae* | *Escherichia coli* | Chromosome | QGR | H-NS |
| stpa_21 | CP069159.1 | StpA | *Enterobacteriaceae* | *Citrobacter sp. R56* | Chromosome | QGR | H-NS |
| ler | BA000007.3 | Ler | *Enterobacteriaceae* | *Escherichia coli O157:H7 str. Sakai* | Chromosome | VGR | Ler |

This table lists the characteristics of representative sequences used to construct the phylogeny of Sfx, H-NS, and StpA homologs. Each sequence is retrieved via tblastn using a seed template (listed in the “Seed” column) from an associated scaffold (“Scaffold accession” column). The taxonomic classification of each scaffold is listed alongside the genomic context (chromosome or plasmid), the AT-hook motif of the homolog (“Motif” column), and the clade classification used in the molecular evolution analysis (See Figure 1A for details).

**Table S2. Characteristics of the 55 representative IncX lineages.**

| Genome Accession (NCBI) | H-NS AT-hook motif | Replicon | Mating pair formation system | Predicted mobility | Predicted host range | GC (%) |
| --- | --- | --- | --- | --- | --- | --- |
| CP025879.1 | RGR | IncX | MPF_T | conjugative | order | 41.1 |
| CP032293.1 | RGR | IncX | MPF_T | conjugative | order | 47.0 |
| CP042617.1 | - | others | - | non-mobilizable | order | 48.4 |
| CP042629.1 | - | IncX, IncF | MPF_F | conjugative | order | 47.9 |
| CP043751.1 | - | others | - | mobilizable | order | 50.5 |
| CP044309.1 | - | IncX, IncF | - | mobilizable | order | 50.5 |
| CP051794.1 | - | IncX | MPF_T | conjugative | family | 41.0 |
| CP054320.1 | RGR | IncX | MPF_T | conjugative | order | 40.7 |
| CP056843.1 | - | IncX, IncF | MPF_F | conjugative | order | 51.9 |
| CP057092.1 | - | IncX, IncF | - | non-mobilizable | order | 46.5 |
| CP057157.1 | - | others | MPF_T | conjugative | family | 39.5 |
| CP057295.1 | - | others | MPF_F | conjugative | order | 51.4 |
| CP057424.1 | - | IncX, IncF | MPF_F | conjugative | order | 47.3 |
| CP066320.1 | - | IncX | - | mobilizable | order | 42.8 |
| CP066331.1 | RGR | IncX | MPF_T | conjugative | order | 41.3 |
| CP074218.1 | RGR | IncX | MPF_T | conjugative | order | 40.5 |
| CP074237.1 | RGR | IncX | MPF_T | conjugative | order | 41.2 |
| LC521833.1 | RGR | others | MPF_T | conjugative | class | 46.4 |
| LC633285.1 | RGR and QGR | others | MPF_F | conjugative | phylum | 51.4 |
| MF554637.1 | - | others | MPF_I | conjugative | order | 49.1 |
| MW264509.1 | NGK | IncX | MPF_T | conjugative | family | 41.8 |
| MW415444.1 | RGR | IncX | MPF_T | conjugative | order | 46.9 |
| NC_010257.1 | RGR | IncX | - | mobilizable | order | 43.5 |
| NC_017624.1 | RGR | IncX | MPF_T | conjugative | order | 41.2 |
| NC_019106.1 | RGR | IncX, IncF | MPF_T | conjugative | order | 48.8 |
| NC_019254.1 | - | IncX, IncF | - | mobilizable | order | 45.8 |
| NZ_CP017233.1 | - | IncX, IncF | MPF_F | conjugative | order | 49.3 |
| NZ_CP022072.1 | - | others | MPF_T | conjugative | phylum | 49.7 |
| NZ_CP022453.1 | - | others | - | non-mobilizable | class | 48.7 |
| NZ_CP023438.1 | RGR | others | MPF_T | conjugative | phylum | 46.8 |
| NZ_CP029439.1 | RGR | IncX | MPF_T | conjugative | order | 48.9 |
| NZ_CP029446.1 | RGR | others | MPF_T | conjugative | phylum | 49.2 |
| NZ_CP029854.1 | RGR | IncX | MPF_T | conjugative | order | 41.7 |
| NZ_CP031548.1 | - | others | MPF_F | conjugative | order | 49.9 |
| NZ_CP031884.1 | RGR | IncX, IncF | MPF_T | conjugative | order | 51.0 |
| NZ_CP032450.1 | RGR and QGR | others | MPF_T | conjugative | phylum | 49.5 |
| NZ_CP054267.1 | RGR | others | MPF_T | conjugative | order | 48.5 |
| NZ_CP056187.1 | RGR | others | MPF_T | conjugative | order | 45.0 |
| NZ_CP060749.1 | - | others | MPF_I | conjugative | order | 53.0 |
| NZ_CP067270.1 | NGK | IncX | MPF_T | conjugative | genus | 40.4 |
| NZ_CP070298.1 | RGR | IncX, IncF | MPF_T | conjugative | order | 42.5 |
| NZ_CP075485.1 | - | IncX, IncF | - | non-mobilizable | order | 49.4 |
| NZ_CP076520.1 | - | IncX, IncF | MPF_F | conjugative | order | 50.0 |
| NZ_CP076649.1 | - | others | - | mobilizable | order | 51.9 |
| NZ_CP076650.1 | VGR | IncX, IncF | MPF_T | conjugative | order | 52.3 |
| NZ_CP077965.1 | IGR | IncX | MPF_T | non-mobilizable | family | 37.9 |
| NZ_CP080228.1 | - | IncX, IncF | MPF_F | mobilizable | order | 47.4 |
| NZ_KX960110.1 | RGR | others | MPF_T | conjugative | order | 51.1 |
| NZ_LT827129.1 | RGR | IncX | MPF_T | conjugative | order | 45.3 |
| NZ_MF150120.1 | RGR | others | MPF_T | conjugative | phylum | 46.1 |
| NZ_MF156712.1 | KGR | others | - | mobilizable | phylum | 47.9 |
| NZ_MK673546.1 | - | IncX, IncF | MPF_F | conjugative | order | 50.9 |
| NZ_MT219819.1 | - | IncX, IncF | - | mobilizable | order | 50.3 |
| NZ_MT219825.1 | RGR and QGR | others | MPF_T | conjugative | order | 46.0 |
| NZ_MT929289.1 | NGK | others | MPF_T | conjugative | family | 42.4 |

Plasmid sequences are retrieved from the PLSDB database and classified using MOB-suite. The sequences are further annotated using PGAP, and orthologous clustering via OrthoFinder is used to identify H-NS homologs. The AT-hook motif of each homolog is listed in the second column, with “-” indicating the absence of a H-NS homolog, and “RGR and QGR” indicating the presence of two H-NS homologs. The listed mobility and host ranges represent MOB-suite predictions.

**Table S3. Likelihood ratio tests from Clade and Branch-site analysis of Sfx, H-NS, and StpA clades.**

| **Model**  **(foreground)** | **np** | **lnL** | **AIC** | **k** | **Parameters** | | | | | **Null** | **LRT** | **df** | **P** |
| --- | --- | --- | --- | --- | --- | --- | --- | --- | --- | --- | --- | --- | --- |
| M2a_rel | 278 | -19133.51 | 38823.02 | 1.763 | **site** | 0 | 1 | 2 |  |  |  |  |  |
|  |  |  |  |  | **proportion** | 0.521 | 0.02 | 0.459 |  |  |  |  |  |
|  |  |  |  |  | **ω** | 0.048 | 1 | 0.188 |  |  |  |  |  |
| CmC alt (Sfx clade) | 279 | -19132.95 | 38823.9 | 1.766 | **site** | 0 | 1 | 2 |  | M2a_rel | 1.119 | 1 | 2.90E-01 |
|  |  |  |  |  | **proportion** | 0.518 | 0.021 | 0.461 |  |  |  |  |  |
|  |  |  |  |  | **background ω** | 0.047 | 1 | 0.193 |  |  |  |  |  |
|  |  |  |  |  | **foreground ω** | 0.047 | 1 | 0.174 |  |  |  |  |  |
| CmC alt (StpA clade) | 279 | -19131.975 | 38821.95 | 1.762 | **site** | 0 | 1 | 2 |  | M2a_rel | 3.07 | 1 | 8.00E-02 |
|  |  |  |  |  | **proportion** | 0.46 | 0.021 | 0.519 |  |  |  |  |  |
|  |  |  |  |  | **background ω** | 0.188 | 1 | 0.051 |  |  |  |  |  |
|  |  |  |  |  | **foreground ω** | 0.188 | 1 | 0.039 |  |  |  |  |  |
| CmC alt (Sfx+ StpA clade) | 280 | -19129.32 | 38818.63 | 1.769 | **site** | 0 | 1 | 2 |  | M2a_rel | 8.388 | 2 | 1.50E-02 |
|  |  |  |  |  | **proportion** | 0.526 | 0.021 | 0.453 |  | Cmc alt (Sfx) | 7.269 | 1 | 7.00E-03 |
|  |  |  |  |  | **background ω** | 0.048 | 1 | 0.174 |  | Cmc alt (StpA) | 5.317 | 1 | 2.10E-02 |
|  |  |  |  |  | **foreground ω (Sfx)** | 0.048 | 1 | 0.176 |  |  |  |  |  |
|  |  |  |  |  | **foreground ω (StpA)** | 0.048 | 1 | 0.231 |  |  |  |  |  |
| CmD alt (Sfx clade) | 280 | -19097.637 | 38755.27 | 1.753 | **site** | 0 | 1 | 2 |  | M2a_rel | 71.747 | 2 | 2.63E-16 |
|  |  |  |  |  | **proportion** | 0.456 | 0.347 | 0.197 |  |  |  |  |  |
|  |  |  |  |  | **background ω** | 0.114 | 0.031 | 0.356 |  |  |  |  |  |
|  |  |  |  |  | **foreground ω** | 0.114 | 0.031 | 0.205 |  |  |  |  |  |
| CmD alt (StpA clade) | 280 | -19089.686 | 38739.37 | 1.767 | **site** | 0 | 1 | 2 |  | M2a_rel | 87.649 | 2 | 9.28E-20 |
|  |  |  |  |  | **proportion** | 0.295 | 0.438 | 0.267 |  |  |  |  |  |
|  |  |  |  |  | **background ω** | 0.026 | 0.098 | 0.214 |  |  |  |  |  |
|  |  |  |  |  | **foreground ω** | 0.026 | 0.098 | 0.439 |  |  |  |  |  |
| CmD alt (H-NS+StpA clade) | 281 | -19087.646 | 38737.29 | 1.767 | **site** | 0 | 1 | 2 |  | M2a_rel | 91.729 | 3 | 9.31E-20 |
|  |  |  |  |  | **proportion** | 0.301 | 0.441 | 0.258 |  | CmD alt (Sfx) | 19.982 | 1 | 7.82E-06 |
|  |  |  |  |  | **background ω** | 0.026 | 0.099 | 0.242 |  | CmD alt (StpA) | 4.08 | 1 | 4.30E-02 |
|  |  |  |  |  | **foreground ω (Sfx)** | 0.026 | 0.099 | 0.189 |  |  |  |  |  |
|  |  |  |  |  | **foreground ω (StpA)** | 0.026 | 0.099 | 0.446 |  |  |  |  |  |
| BrS null (ancestral Sfx branch) | 277 | -19342.363 | 39238.73 | 1.756 | **site** | 0 | 1 | 2a | 2b |  |  |  |  |
|  |  |  |  |  | **proportion** | 0.701 | 0.038 | 0.248 | 0.013 |  |  |  |  |
|  |  |  |  |  | **background ω** | 0.105 | 1 | 0.105 | 1 |  |  |  |  |
|  |  |  |  |  | **foreground ω** | 0.105 | 1 | 1 | 1 |  |  |  |  |
| BrS alt (ancestral Sfx branch) | 278 | -19339.408 | 39234.82 | 1.769 | **site** | 0 | 1 | 2a | 2b | BrS null (Sfx) | 5.91 | 1 | 1.50E-02 |
|  |  |  |  |  | **proportion** | 0.738 | 0.039 | 0.211 | 0.011 |  |  |  |  |
|  |  |  |  |  | **background ω** | 0.105 | 1 | 0.105 | 1 |  |  |  |  |
|  |  |  |  |  | **foreground ω** | 0.105 | 1 | 11.423 | 11.423 |  |  |  |  |

PAML 4.10.6 is used to fit the Clade (CmC, CmD) and Branch-site (BrS) models. For models used to test for positive selection (“alt”), sites with divergent ratios of nonsynonymous to synonymous substitution rates (ω) are not fixed (Clade model) or are forced to be above 1 (Branch-site model). For all null models (“null”), the divergent ω is fixed to 1. The Chi-square test is used to derive the *p*-values. Abbreviations: np (number of parameters); lnL (log-likelihood score); AIC (Akaike information criterion); k (kappa; transition:transversion rate ratio); null (null model); LRT (likelihood ratio test); df (degrees of freedom); P (*p*-value); site (site class shorthand); proportion (proportion of alignment sites that fall into site class); ω (ratios of nonsynonymous to synonymous substitution rates).

**Table S4. Strains used in the study.**

| **Strain** | **Description** | **Source** |
| --- | --- | --- |
| DH5α | Cloning and plasmid propagation *E. coli* strain | NEB |
| BTH101 | *E. coli* *cya* mutant for use in bacterial-two-hybrid assays | Euromedex |
| EcoR25 | Spontaneous NaN_3_^r^ derivative of EcoR25 | This work |
| BW25113 | Parent strain for the Keio collection | (1) |
| BW25113∆*hns* | *E. coli* BW25113 with *hns* deleted and replaced with a kanR casette | (1) |
| BW25113∆*stpA* | *E. coli* BW25113 with *stpA* deleted and replaced with a kanR casette | (1) |
| BW25113∆*hha* | *E. coli* BW25113 with *hha* deleted and replaced with a kanR casette | (1) |
| BW25113∆*cnu* | *E. coli* BW25113 with *cnu* deleted and replaced with a kanR casette | (1) |
| EC6 | *E. coli* BW25113 carrying wildtype R6K | This work |
| EC12 | *E. coli* BW25113∆*hns* carrying wildtype R6K | This work |
| EC13 | *E. coli* BW25113∆*hns* carrying R6K∆*sfx* | This work |
| EC7 | *E. coli* BW25113 carrying R6K∆*sfx* | This work |
| EC15 | *E. coli* BW25113 carrying R6K∆*sfx* and pHSG576 empty vector | This work |
| EC16 | *E. coli BW25113∆hns carrying pHSG576 empty vector* | This work |
| EC25 | *E. coli* BW25113∆*hns* carrying pAV2 (pHSG576::*hns* (endogenous *hns* promoter)) | This work |
| EC27 | *E. coli* BW25113∆*hns* carrying pAV6 (pHSG576::*sfx* (endogenous *sfx* promoter)) | This work |
| EC29 | *E. coli* BW25113 carrying R6K∆*sfx* and pAV2 (pHSG576::*hns* (endogenous *hns* promoter)) | This work |
| EC31 | *E. coli* BW25113 carrying R6K∆*sfx* and pAV6 (pHSG576::*sfx* (endogenous *sfx* promoter)) | This work |
| EC45 | *E. coli* BW25113 carrying R6K∆*sfx* and pAV8 (pHSG576::*hns*(XHH)) | This work |
| EC46 | *E. coli* BW25113 carrying R6K∆*sfx* and pAV9 (pHSG576::*hns*(HHX)) | This work |
| EC47 | *E. coli* BW25113 carrying R6K∆*sfx* and pAV10 (pHSG576::*hns*(HXH)) | This work |
| EC48 | *E. coli* BW25113 carrying R6K∆*sfx* and pAV11 (pHSG576::*hns*(XXH)) | This work |
| EC49 | *E. coli* BW25113 carrying R6K∆*sfx* and pAV12 (pHSG576::*hns*(HXX)) | This work |
| EC50 | *E. coli* BW25113 carrying R6K∆*sfx* and pAV13 (pHSG576::*hns*(XHX)) | This work |
| EC51 | *E. coli* BW25113 carrying R6K∆*sfx* and pAV14 (pHSG576::*sfx*_N-truncated)) | This work |
| EC52 | *E. coli* BW25113 carrying R6K∆*sfx* and pAV15 (pHSG576::*sfx*_C-truncated) | This work |
| EC53 | *E. coli* BW25113 carrying R6K∆*sfx* and pAV16 (pHSG576::*sfx*-QGR) | This work |
| EC63 | *E. coli* BW25113 carrying R6K∆*sfx* and pAV17 (pHSG576::*hns*(XXH)-coil1X) | This work |
| EC64 | *E. coli* BW25113 carrying R6K∆*sfx* and pAV*18* (pHSG576::*hns*(XXH)-coil2X) | This work |
| EC65 | *E. coli* BW25113 carrying R6K∆*sfx* and pAV19 (pHSG576::*hns*(XXH)-Q112R) | This work |
| EC88 | *E. coli* BW25113 carrying R6K∆*sfx* and pAV22 (pHSG576:*:hns-sfx*P (endogenous Sfx promoter)) | This work |
| EC89 | *E. coli* BW25113 carrying R6K∆*sfx* and pAV23 (pHSG576::*hns-sfx*P-coil1X (endogenous Sfx promoter)) | This work |
| EC90 | *E. coli* BW25113 carrying R6K∆*sfx* and pAV24 (pHSG576::*hns-sfx*P-coil2X (endogenous Sfx promoter)) | This work |
| EC91 | *E. coli* BW25113 carrying R6K∆*sfx* and pAV25 (pHSG576::*hns-sfx*P-Q112R (endogenous Sfx promoter)) | This work |
| EC94 | *E. coli* BW25113∆*hha* carrying R6K | This work |
| EC95 | *E. coli* BW25113∆*hha* carrying R6K∆*sfx* | This work |
| EC96 | *E. coli* BW25113∆*cnu* carrying R6K | This work |
| EC97 | *E. coli* BW25113∆*cnu* carrying R6K∆*sfx* | This work |
| EC98 | *E. coli* BW25113∆*stpA* carrying R6K | This work |
| EC99 | *E. coli* BW25113∆*stpA* carrying R6K∆*sfx* | This work |
| EC107 | *E. coli* BW25113∆*hha* carrying R6K and pHSG576 empty vector | This work |
| EC108 | *E. coli* BW25113∆*hha* carrying R6K and pAV52 (pHSG576::*hha* (endogenous *hha* vector)) | This work |
| B2H_32_EV | *E. coli* BTH101 carrying pAV32 (pUT18::*hns* (M1-G85)) and pKNT25 | This work |
| B2H_32_26 | *E. coli* BTH101 carrying pAV32 (pUT18::*hns* (M1-G85)) and pAV26 (pKNT25::*hns)* | This work |
| B2H_32_27 | *E. coli* BTH101 carrying pAV32 (pUT18::*hns* (M1-G85)) and pAV27 (pKNT25::*stpA*) | This work |
| B2H_32_28 | *E. coli* BTH101 carrying pAV32 (pUT18::*hns* (M1-G85)) and pAV28 (pKNT25::*sfx*) | This work |
| B2H_32_29 | *E. coli* BTH101 carrying pAV32 (pUT18::*hns* (M1-G85)) and pAV29 (pKNT25::*sfx*_N-truncated) | This work |
| B2H_32_30 | *E. coli* BTH101 carrying pAV32 (pUT18::*hns* (M1-G85)) and pAV30 (pKNT25::*hha*) | This work |
| B2H_32_31 | *E. coli* BTH101 carrying pAV32 (pUT18::*hns* (M1-G85)) and pAV31 (pKNT25::*cnu*) | This work |
| B2H_32_38 | *E. coli* BTH101 carrying pAV32 (pUT18::*hn*s (M1-G85)) and pAV38 (pKT25::*hha)* | This work |
| B2H_32_39 | *E. coli* BTH101 carrying pAV32 (pUT18::*hns* (M1-G85)) and pAV39 (pKT25::*cnu*) | This work |
| B2H_33_EV | *E. coli* BTH101 carrying pAV33 (pUT18:*:stpA*) and pKNT25 | This work |
| B2H_33_26 | *E. coli* BTH101 carrying pAV33 (pUT18::*stpA*) and pAV26 (pKNT25::*hns)* | This work |
| B2H_33_27 | *E. coli* BTH101 carrying pAV33 (pUT18::*stpA*) and pAV27 (pKNT25::*stpA*) | This work |
| B2H_33_28 | *E. coli* BTH101 carrying pAV33 (pUT18::*stpA*) and pAV28 (pKNT25::*sfx*) | This work |
| B2H_33_29 | *E. coli* BTH101 carrying pAV33 (pUT18::*stpA*) and pAV29 (pKNT25::*sfx*_N-truncated) | This work |
| B2H_33_30 | *E. coli* BTH101 carrying pAV33 (pUT18::*stpA*) and pAV30 (pKNT25::*hha*) | This work |
| B2H_33_31 | *E. coli* BTH101 carrying pAV33 (pUT18::*stpA*) and pAV31 (pKNT25::*cnu*) | This work |
| B2H_33_38 | *E. coli* BTH101 carrying pAV33 (pUT18::*stpA*) and pAV38 (pKT25::*hha)* | This work |
| B2H_33_39 | *E. coli* BTH101 carrying pAV33 (pUT18::*stpA*) and pAV39 (pKT25::*cnu*) | This work |
| B2H_34_EV | *E. coli* BTH101 carrying pAV34 (pUT18::*sfx*) and pKNT25 | This work |
| B2H_34_26 | *E. coli* BTH101 carrying pAV34 (pUT18::*sfx*) and pAV26 (pKNT25::*hns)* | This work |
| B2H_34_27 | *E. coli* BTH101 carrying pAV34 (pUT18::*sfx*) and pAV27 (pKNT25::*stpA*) | This work |
| B2H_34_28 | *E. coli* BTH101 carrying pAV34 (pUT18::*sfx*) and pAV28 (pKNT25::*sfx*) | This work |
| B2H_34_29 | *E. coli* BTH101 carrying pAV34 (pUT18::*sfx*) and pAV29 (pKNT25::*sfx*_N-truncated) | This work |
| B2H_34_30 | *E. coli* BTH101 carrying pAV34 (pUT18::*sfx*) and pAV30 (pKNT25::*hha*) | This work |
| B2H_34_31 | *E. coli* BTH101 carrying pAV34 (pUT18::*sfx*) and pAV31 (pKNT25::*cnu*) | This work |
| B2H_34_38 | *E. coli* BTH101 carrying pAV34 (pUT18::*sfx*) and pAV38 (pKT25::*hha)* | This work |
| B2H_34_39 | *E. coli* BTH101 carrying pAV34 (pUT18::*sfx*) and pAV39 (pKT25::*cnu*) | This work |
| B2H_35_EV | *E. coli* BTH101 carrying pAV35 (pUT18::*sfx-*N-truncated) and pKNT25 | This work |
| B2H_35_26 | *E. coli* BTH101 carrying pAV35 (pUT18::*sfx*-N-truncated) and pAV26 (pKNT25::*hns)* | This work |
| B2H_35_27 | *E. coli* BTH101 carrying pAV35 (pUT18::*sfx*-N-truncated) and pAV27 (pKNT25::*stpA*) | This work |
| B2H_35_28 | *E. coli* BTH101 carrying pAV35 (pUT18::*sfx*-N-truncated) and pAV28 (pKNT25::*sfx*) | This work |
| B2H_35_29 | *E. coli* BTH101 carrying pAV35 (pUT18::*sfx*-N-truncated) and pAV29 (pKNT25::*sfx*_N-truncated) | This work |
| B2H_35_30 | *E. coli* BTH101 carrying pAV35 (pUT18::*sfx*-N-truncated)) and pAV30 (pKNT25::*hha*) | This work |
| B2H_35_31 | *E. coli* BTH101 carrying pAV35 (pUT18::*sfx*-N-truncated) and pAV31 (pKNT25::*cnu*) | This work |
| B2H_35_38 | *E. coli* BTH101 carrying pAV35 (pUT18::*sfx*-N-truncated) and pAV38 (pKT25::*hha)* | This work |
| B2H_35_39 | *E. coli* BTH101 carrying pAV35 (pUT18::*sfx*-N-truncated) and pAV39 (pKT25::*cnu*) | This work |
| B2H_40_EV | *E. coli* BTH101 carrying pAV40 (pUT18C::*hha*) and pKNT25 | This work |
| B2H_40_26 | *E. coli* BTH101 carrying pAV40 (pUT18C::*hha*) and pAV26 (pKNT25::*hns)* | This work |
| B2H_40_27 | *E. coli* BTH101 carrying pAV40 (pUT18C::*hha*) and pAV27 (pKNT25::*stpA*) | This work |
| B2H_40_28 | *E. coli* BTH101 carrying pAV40 (pUT18C::*hha*) and pAV28 (pKNT25::*sfx*) | This work |
| B2H_40_29 | *E. coli* BTH101 carrying pAV40 (pUT18C::*hha*) and pAV29 (pKNT25::*sfx*_N-truncated) | This work |
| B2H_40_30 | *E. coli* BTH101 carrying pAV40 (pUT18C::*hha*) and pAV30 (pKNT25::*hha*) | This work |
| B2H_40_31 | *E. coli* BTH101 carrying pAV40 (pUT18C::*hha*) and pAV31 (pKNT25::*cnu*) | This work |
| B2H_40_38 | *E. coli* BTH101 carrying pAV40 (pUT18C::*hha*) and pAV38 (pKT25::*hha)* | This work |
| B2H_40_39 | *E. coli* BTH101 carrying pAV40 (pUT18C::*hha*) and pAV39 (pKT25::*cnu*) | This work |
| B2H_41_EV | *E. coli* BTH101 carrying pAV41 (pUT18C::*cnu*) and pKNT25 | This work |
| B2H_41_26 | *E. coli* BTH101 carrying pAV41 (pUT18C::*cnu*) and pAV26 (pKNT25::*hns)* | This work |
| B2H_41_27 | *E. coli* BTH101 carrying pAV41 (pUT18C::*cnu*) and pAV27 (pKNT25::*stpA*) | This work |
| B2H_41_28 | *E. coli* BTH101 carrying pAV41 (pUT18C::*cnu*) and pAV28 (pKNT25::*sfx*) | This work |
| B2H_41_29 | *E. coli* BTH101 carrying pAV41 (pUT18C::*cnu*) and pAV29 (pKNT25::*sfx*_N-truncated) | This work |
| B2H_41_30 | *E. coli* BTH101 carrying pAV41 (pUT18C:*:cnu*) and pAV30 (pKNT25::*hha*) | This work |
| B2H_41_31 | *E. coli* BTH101 carrying pAV41 (pUT18C::cnu) and pAV31 (pKNT25::*cnu*) | This work |
| B2H_41_38 | *E. coli* BTH101 carrying pAV41 (pUT18C::*cnu*) and pAV38 (pKT25::*hha)* | This work |
| B2H_41_39 | *E. coli* BTH101 carrying pAV41 (pUT18C:*:cnu*) and pAV39 (pKT25::*cnu*) | This work |

**Table S5. Plasmids used in the study.**

| **Plasmid ID** | **Description** | **Source** |
| --- | --- | --- |
| R6K | IncX2 model plasmid | (2) |
| R6K∆*sfx* | R6K with *sfx* deleted and replaced with a kanR casette | This work |
| pHSG576 | Low-copy expression vector (Cm^r^) | (3) |
| pUT18 | High-copy vector containing T18 fragment (Amp^r^) | Euromedex |
| pUT18C | High-copy vector containing T18 fragment (Amp^r^) | Euromedex |
| pKNT25 | Low-copy vector containing T25 fragment (Kan^r^) | Euromedex |
| pKT25 | Low-copy vector containing T25 fragment (Kan^r^) | Euromedex |
| pAV2 | pHSG576 containing *hns* from E. coli BW25113 expressed from its native promoter | This work |
| pAV6 | pHSG576 containing *sfx* from R6K expressed from its native promoter | This work |
| pAV8 | pHSG576 encoding for a chimeric H-NS protein expressed from the endogenous *sfx* promoter. Chimeric H-NS (XHH) consists of M1-R47 (from Sfx) and E42-Q137 (from *E. coli* H-NS) | This work |
| pAV9 | pHSG576 encoding for a chimeric H-NS protein expressed from the endogenous *sfx* promoter. Chimeric H-NS (HHX) consists of M1-G85 (*E. coli* H-NS), F84-A149 (R6K Sfx) | This work |
| pAV10 | pHSG576 encoding for a chimeric H-NS protein expressed from the endogenous *sfx* promoter. Chimeric H-NS (HXH) consists of M1-R41 (*E. coli* H-NS), E48-G83 (R6K Sfx), and T86-Q137 (*E. coli* H-NS) | This work |
| pAV11 | pHSG576 encoding for a chimeric H-NS protein expressed from the endogenous *sfx* promoter. Chimeric H-NS (XXH) consists of M1-G83 (R6K Sfx) and T86-Q137 (*E. coli* H-NS) | This work |
| pAV12 | pHSG576 encoding for a chimeric H-NS protein expressed from the endogenous *sfx* promoter. Chimeric H-NS (HXX) consists of M1-R41 (*E. coli* H-NS) and E48-A149 (R6K Sfx) | This work |
| pAV13 | pHSG576 encoding for a chimeric H-NS protein expressed from the endogenous *sfx* promoter. Chimeric H-NS (XHX) consists of M1-R47 (R6K Sfx), E42-G85 (*E. coli* H-NS), and F84-A149 (R6K Sfx) | This work |
| pAV14 | pHSG576 encoding for Sfx with N-terminus truncated (∆D3-E8). Expressed from endogenous *sfx* promoter | This work |
| pAV15 | pHSG576 encoding for Sfx with C-terminus truncated (∆K141-A149). Expressed from endogenous *sfx* promoter | This work |
| pAV16 | pHSG576 expressing Sfx (R117Q) from its endogenous *sfx* promoter. | This work |
| pAV17 | pHSG576 encoding for a chimeric H-NS protein expressed from the endogenous *sfx* promoter. Chimeric H-NS (XXH-coil 1), consists of pAV11 with coil 1 (T86-K96) replaced with coil 1 from Sfx (F84-K102) | This work |
| pAV18 | pHSG576 encoding for a chimeric H-NS protein expressed from the endogenous *sfx* promoter. Chimeric H-NS (XXH-coil 2), consists of pAV11 with coil 2 (T110-P116) replaced with coil 1 from Sfx (S115-P121) | This work |
| pAV19 | pHSG576 encoding for a chimeric H-NS protein expressed from the endogenous *sfx* promoter. Chimeric H-NS (XXH-coil 2) consists of pAV11 with its QGR changed to an RGR-AT hook motif (Q112R) | This work |
| pAV22 | pHSG576 encoding for *E. coli* H-NS expressed from the endogenous *sfx* promoter | This work |
| pAV23 | pHSG576 encoding for a chimeric H-NS protein expressed from the endogenous *sfx* promoter. Chimeric H-NS (H-NS-coil 1), consists of pAV22 with coil 1 (T86-K96) replaced with coil 1 from Sfx (F84-K102) | This work |
| pAV24 | pHSG576 encoding for a chimeric H-NS protein expressed from the endogenous *sfx* promoter. Chimeric H-NS (H-NS-coil 2), consists of pAV22 with coil 2 (T110-P116) replaced with coil 1 from Sfx (S115-P121) | This work |
| pAV25 | pHSG576 encoding for *E. coli* H-NS (Q112R) expressed from the endogenous *sfx* promoter | This work |
| pAV26 | pKNT25::*hns* | This work |
| pAV27 | pKNT25::*stpA* | This work |
| pAV28 | pKNT25:*:sfx* | This work |
| pAV29 | pKNT25::*sfx*_N-truncated (∆D3-E8) | This work |
| pAV30 | pKNT25::*hha* | This work |
| pAV31 | pKNT25::*cnu* | This work |
| pAV32 | pUT18::*hns*(M1-G85) | This work |
| pAV33 | pUT18:*:stpA* | This work |
| pAV34 | pUT18::*sfx* | This work |
| pAV35 | pUT18::*sfx*_N-truncated (∆D3-E8) | This work |
| pAV36 | pUT18::*hha* | This work |
| pAV37 | pUT18::*cnu* | This work |
| pAV38 | pKT25::*hha* | This work |
| pAV39 | pKT25::*cnu* | This work |
| pAV40 | pUT18C::*hha* | This work |
| pAV41 | pUT18C::*cnu* | This work |
| pAV52 | pHSG576::hha | This work |

**Table S6. Oligonucleotides used in the study.**

| **Name** | **Sequence** |
| --- | --- |
| AV5_linear_pWN423_FLAG_F | gactacaaggacgacgatgac |
| AV6_linear_pWN423_FLAG_R | tgcaggtcgacggatcc |
| AV8_gs_hnsec_CFLAG_R | ggatccgtcgacctgcacagccacaggccctcaatg |
| AV9_gs_hnsec_F | gtcatcgtcgtccttgtagtcttattgcttgatcaggaaatcgtcg |
| AV12_gs_hnsx_F | ggatccgtcgacctgcattcaaactctggtcagaaattgctg |
| AV14_gs_hnsx_R | gtcatcgtcgtccttgtagtcttatgcttgctcggtgcc |
| AV27_sdm_hnsx-promoter_F | agtcaatgcccttattttttagcga |
| AV28_sdm_hnsx-promoter_R | gactacaaggacgacgatgaca |
| AV29_gs_hnsx-D1_R | agccgcgctttcttcttctcggcgctcttcaataacag |
| AV30_gs_hns-D2_F | ctgttattgaagagcgccgagaagaagaaagcgcggctg |
| AV31_gs_hns-D1_F | gctaaaaaataagggcattgactatgagcgaagcacttaaaattctga |
| AV32_gs_hns-D2_R | gttttttcggtttctcttcgctaaagccagatttaacggcagcaag |
| AV33_gs_hnsx-D3_F | cttgctgccgttaaatctggctttagcgaagagaaaccgaaaaaac |
| AV34_gs_hns-D1_R | ggcttcctgttcagcttcttcgcgacgttcgttaacaacaac |
| AV35_gs_hnsx-D2_F | gttgttgttaacgaacgtcgcgaagaagctgaacaggaagcc |
| AV36_gs_hnsx-D2_R | ctgagcacgtttagctttggttcctagtagttcttcagcagagaaac |
| AV37_gs_hns-D3_F | gtttctctgctgaagaactactaggaaccaaagctaaacgtgctcag |
| AV38_sdm_hnsx-Nterm_R | actcatagtcaatgcccttattttttagc |
| AV39_sdm_hnsx_Nterm_F | ttagtgcgtaagcttcttttaaacatc |
| AV40_sdm_hnsx_Cterm_R | ttgcttgatgagaaaatcttcaagcttctg |
| AV41_sdm_hnsx_Cterm_F | taagactacaaggacgacgatgac |
| AV42_sdm_hnsx_QGR_F | gcgcctaaaccaatcgatgc |
| AV43_sdm_hnsx_QGR_R | acgaccttgaccagaccagt |
| AV44_sdm_hns-hnsx-coil1_F | aaaaacaaacttccaaaagccccgccaaaatatagctacgttgac |
| AV45_sdm_hns-hnsx-coil1_R | gcgttttttcggtttctcttcgctaaatcctagtagttcttcagcagagaaac |
| AV46_sdm_hns-RGR_F | ctggccgcggccgtactcc |
| AV47_sdm_hns-RGR_R | tccaggttttagtttcgccgt |
| AV48_sdm_hns_R93A_F | ctcaggccccggcaaaatatagc |
| AV49_sdm_hns_R93A_R | cacgtttagctttggttcctagtag |
| AV50_sdm_hns-hnsx_coil2_F | ggtcgtgcgccagctgtaatcaa |
| AV51_sdm_hns-hnsx_coil2_R | gcgaccagaccaggttttagtttcgc |
| AV52_cp_hnsx-coil1_F | ccaaaagccccgccaaaa |
| AV53_cp_hnsx-coil2_F | gaaactaaaacctggtctggtcg |
| AV54_linear_pKNT25_pUT18_F | accatgattacgccaagcttg |
| AV55_linear_pKNT25_pUT18_R | agctgtttcctgtgtgaaattgttatc |
| AV56_gs_hns_pKNT25_pUT18_F | caatttcacacaggaaacagctatgagcgaagcacttaaaattctg |
| AV57_gs_hns_pKNT25_pUT18_R | gcaagcttggcgtaatcatggtttgcttgatcaggaaatcgtcg |
| AV58_gs_stpa_pKNT25_pUT18_F | caatttcacacaggaaacagctatgtccgtaatgttacaaagtttaaataacattc |
| AV59_gs_stpa_pKNT25_pUT18_R | gcaagcttggcgtaatcatggtgatcaggaaatcgtcgagagatttac |
| AV60_gs_hnsx_pKNT25_pUT18_F | caatttcacacaggaaacagctatgagtgataatgaaaattttgaattagtgc |
| AV61_gs_hnsx_pKNT25_pUT18_R | gcaagcttggcgtaatcatggttgcttgctcggtgcc |
| AV62_gs_hnsx-Ntrunc_pKNT25_pUT18_F | caatttcacacaggaaacagctatgagtttagtgcgtaagcttcttttaa |
| AV63_gs_hha_pKNT25_pUT18_F | caatttcacacaggaaacagctatgtccgaaaaacctttaacgaaaac |
| AV64_gs_hha_pKNT25_pUT18_R | gcaagcttggcgtaatcatggtgcgaataaatttccatactgaggaag |
| AV65_gs_ydgt_pKNT25_pUT18_F | caatttcacacaggaaacagctatgactgttcaggactacttattaaaatttcg |
| AV66_gs_ydgt_pKNT25_pUT18_R | gcaagcttggcgtaatcatggtttggacatagtgccagacgg |
| AV67_linear_pKT25_F | gtaagaattcactggccgtcg |
| AV68_linear_pKT25_R | cttaggtacccggggatcc |
| AV69_linear_pUT18C_F | ctaagtaatatggtgcactctcagtac |
| AV70_linear_pUT18C_R | tatcgatgaattcgagctcggtac |
| AV71_gs_hha_pKT25_F | gaggatccccgggtacctaagtccgaaaaacctttaacgaaaacc |
| AV72_gs_hha_pKT25_R | cgacggccagtgaattcttacttagcgaataaatttccatactgaggaag |
| AV73_gs_hha_pUT18C_F | ccgagctcgaattcatcgatatccgaaaaacctttaacgaaaacc |
| AV74_gs_hha_pUT18C_R | gagagtgcaccatattacttagttagcgaataaatttccatactgaggaag |
| AV75_gs_ydgt_pKT25_F | gaggatccccgggtacctaagactgttcaggactacttattaaaatttcgc |
| AV76_gs_ydgt_pKT25_R | cgacggccagtgaattcttacttattggacatagtgccagacgg |
| AV77_gs_ydgt_pUT18C_F | ccgagctcgaattcatcgataactgttcaggactacttattaaaatttcgc |
| AV78_gs_ydgt_pUT18C_R | gagagtgcaccatattacttagttattggacatagtgccagacgg |
| AV85_sdm_hns-hnsx-coil1-v2_R | gcgttttttcggtttctcttcgctaaagccagatttaacggcagcaa |
| AV86_gs_hns-D2_pUT18_R | gcaagcttggcgtaatcatggtgccagatttaacggcagcaag |
| AV98_gs_hha_F | ggatccgtcgacctgcatgaagacaataagctcattgagcag |
| AV99_gs_hha_R | gtcatcgtcgtccttgtagtcttagcgaataaatttccatactgaggaag |

**References**

1. Baba T, Ara T, Hasegawa M, Takai Y, Okumura Y, Baba M, Datsenko KA, Tomita M, Wanner BL, Mori H. 2006. Construction of *Escherichia coli* K-12 in-frame, single-gene knockout mutants: the Keio collection. Mol Syst Biol 2:2006.0008.

2. Kontomichalou P, Mitani M, Clowes RC. 1970. Circular R-Factor Molecules Controlling Penicillinase Synthesis, Replicating in *Escherichia coli* Under Either Relaxed or Stringent Control. Journal of Bacteriology 104:34–44.

3. Takeshita S, Sato M, Toba M, Masahashi W, Hashimoto-Gotoh T. 1987. High-copy-number and low-copy-number plasmid vectors for *lacZα*-complementation and chloramphenicol- or kanamycin-resistance selection. Gene 61:63–74.
