# Supplementary material for: Evolutionary and functional divergence of Sfx, a plasmid-encoded H-NS homolog, underlies the regulation of IncX plasmid conjugation
Source: mBio. 2024 Dec 23;16(2):e02089-24. doi: 10.1128/mbio.02089-24 (PMC11796372; doi:10.1128/mbio.02089-24)
Supplement: Supplemental Figures — Figures S1 to S5. [file mbio.02089-24-s0002.docx]

**Supplementary Figures**

**
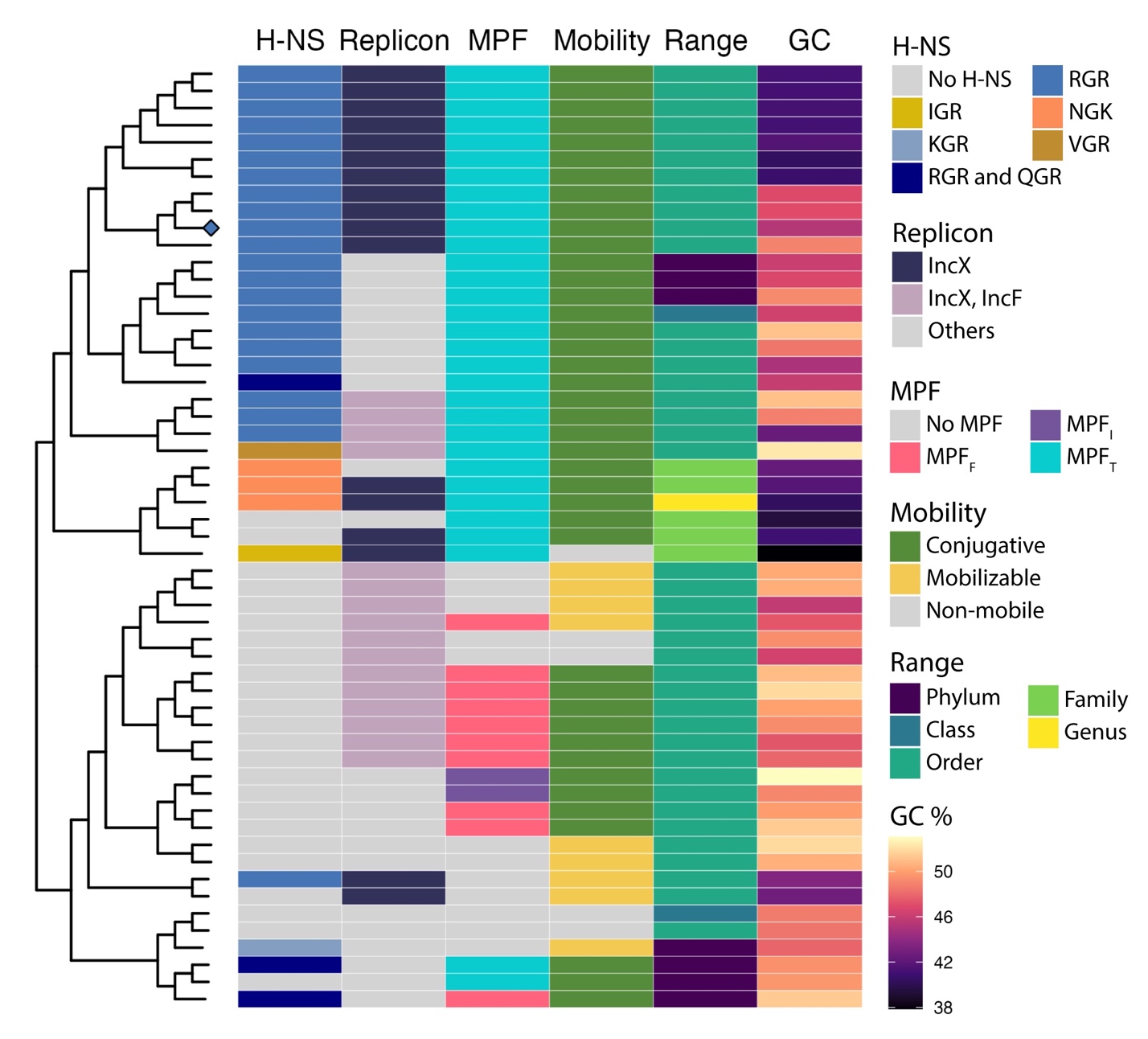
Figure S1. MPF_T_-type IncX plasmids frequently carry Sfx homologs.** 55 representative IncX lineages were analyzed using orthologous clustering and MOB-Typer to identify H-NS homologs and characterize plasmid attributes. The plasmids are clustered using hierarchical clustering based on various attributes, such as the AT-hook motif of the H-NS homolog (“H-NS"), predicted replicon type (“Replicon”), MPF (mating pair formation) type, predicted mobility (“Mobility”), predicted host range (“Range”), and GC%. Note that plasmids with a H-NS label of “RGR and QGR” carry two H-NS homologs, one with a RGR AT-hook motif and the other with a QGR AT-hook motif. The node representing R6K is marked with a blue diamond.


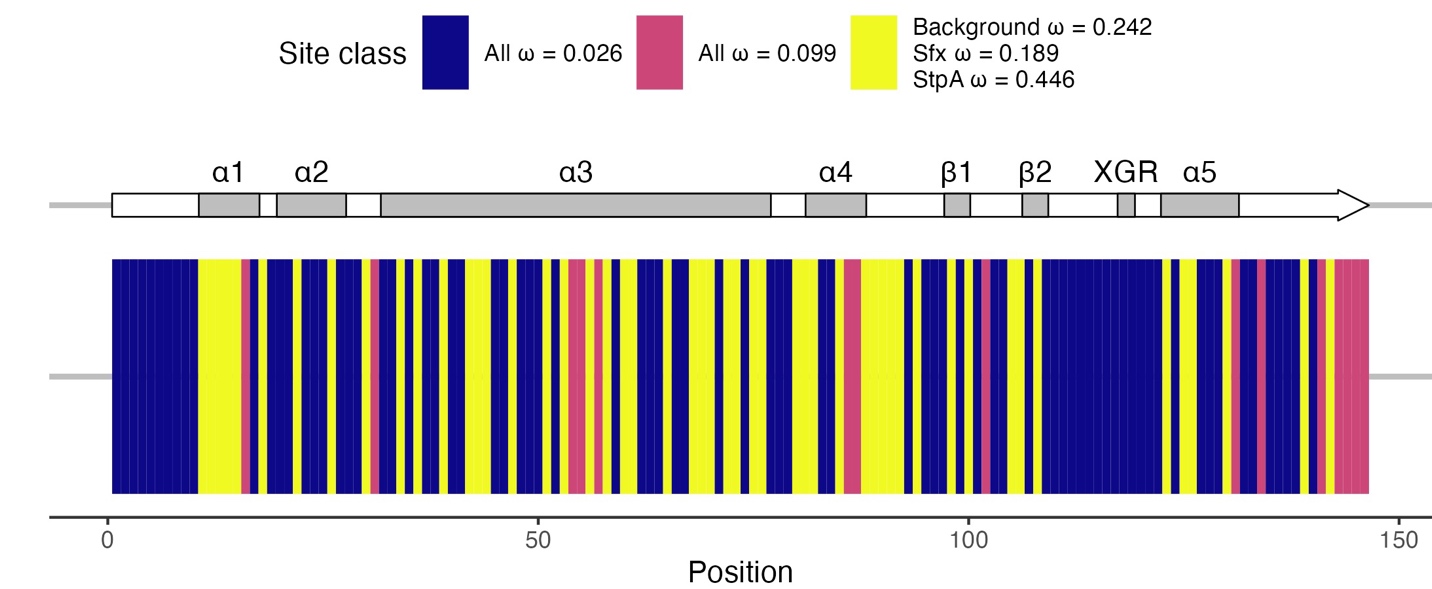


**Figure S2. The StpA clade exhibits less selective constraint.** The gene map is colored based on the site classification derived from Bayes Empirical Bayes (BEB) analysis of the best performing multi-clade CmD alternative model (Sfx and StpA clades set as foregrounds). 3 site classes are listed, each representing a specific ratio of nonsynonymous substitution rates to synonymous substitution rates (ω). Blue squares represent sites under the highest degree of purifying selection followed by the magenta squares (neutrally evolving sites). The yellow squares represent sites under different selective constraints across the Sfx, StpA, and H-NS clades. The corresponding secondary structure at each alignment position is shown above the heatmap and is derived from a Colabfold-predicted structure of *E. coli* K12 H-NS (this is used instead of Sfx due to gaps in the alignment of the linker region). Abbreviations: α (alpha-helix); β (beta-sheet).


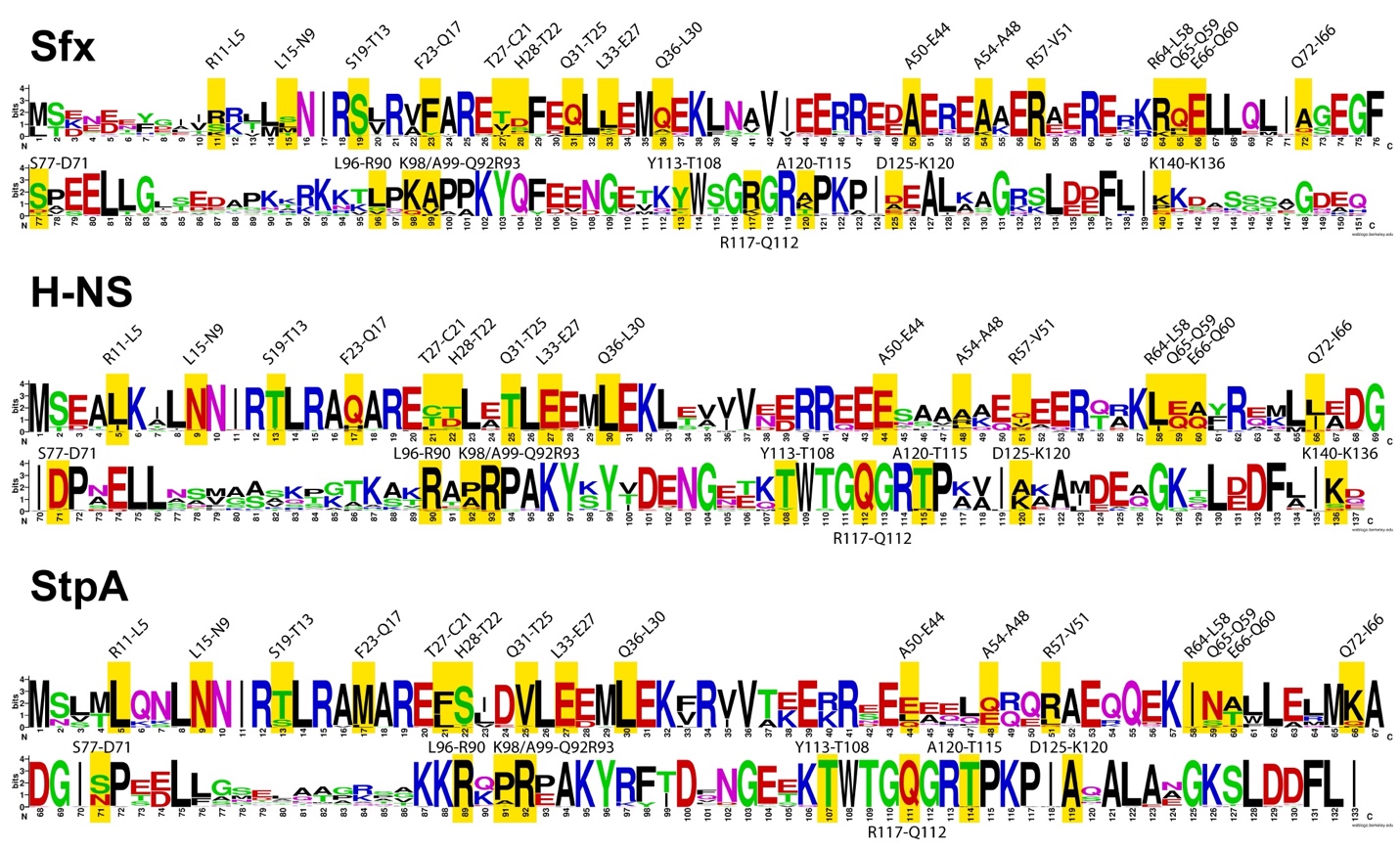


**Figure S3. ­Positively selected residue positions are mostly conserved within phylogenetic clades.** Sequences within the Sfx, H-NS, and StpA clades (See Figure 1 for details) are aligned using MAFFT E-INS-i. The conservation of amino acids at each position is visualized using WebLogo. The locations of positively selected sites, as calculated by Naive Empirical Bayes and Bayes Empirical Bayes analysis, are highlighted in yellow. The amino acid transitions from Sfx (R6K) to *E. coli* K12 H-NS are printed above each position.

**
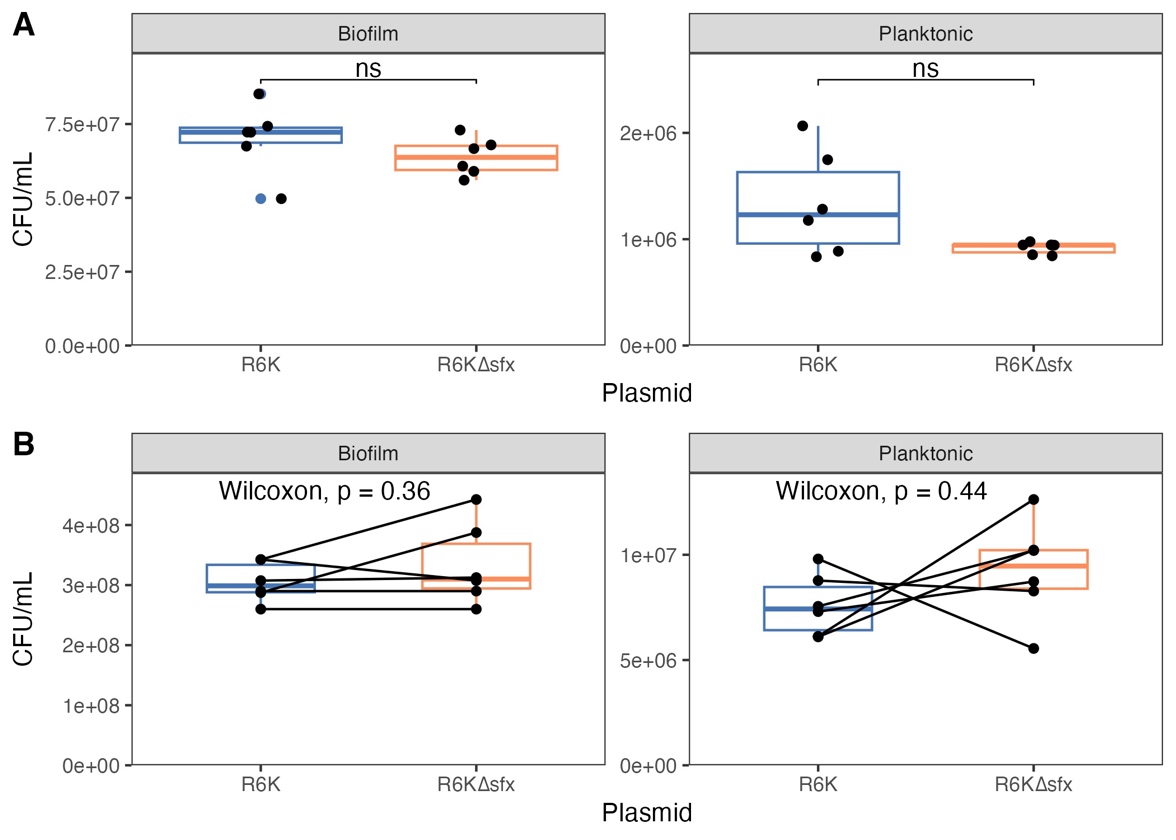
**

**Figure S4. Sfx loss does not affect R6K carrier growth in biofilms.** (A) Sfx loss does not affect R6K donor growth in a biofilm conjugation setting. Plotted on the y-axis are the final donor population densities of R6K (left) and R6K∆*sfx* donors in the biofilm (left panel) and planktonic (right panel) states. Briefly, R6K/R6K∆*sfx* donors and EcoR25 recipients (NaN_3_^R^) are seeded at a starting OD_600_ of 0.03 and cultured statically at 30˚C in 150 µL of M9+0.2% glucose media in a 96-well polystyrene plate. The final population density of donor, transconjugant, and recipient cells after 24 hours is assessed by selective plating. Statistical significance is assessed using the Student’s t-test with Benjamini-Hochberg correction. (B) R6K and R6K∆*sfx* carriers have similar growth rates in a biofilm setting. Each jointed point represents the final population density of R6K (left) and R6K∆*sfx* (right) carriers in the biofilm (left panel) and planktonic (right panel) states. The competition assay is performed by inoculating R6K and R6K∆*sfx* carriers to a final OD_600_ of 0.03 and coculturing them at 30˚C in 150 µL of M9+0.2% glucose media in a 96-well polystyrene plate. The final population composition of R6K and R6K∆*sfx* carriers after 24 hours of incubation is assessed using selective plating. Statistical significance is assessed using the Wilcoxon matched pairs test. (A-B) displays the pooled results from two experimental replicates, with each point representing a biological replicate (averaged value across two technical replicates). Abbreviations: ns (adjusted *p-*value ≥ 0.05).


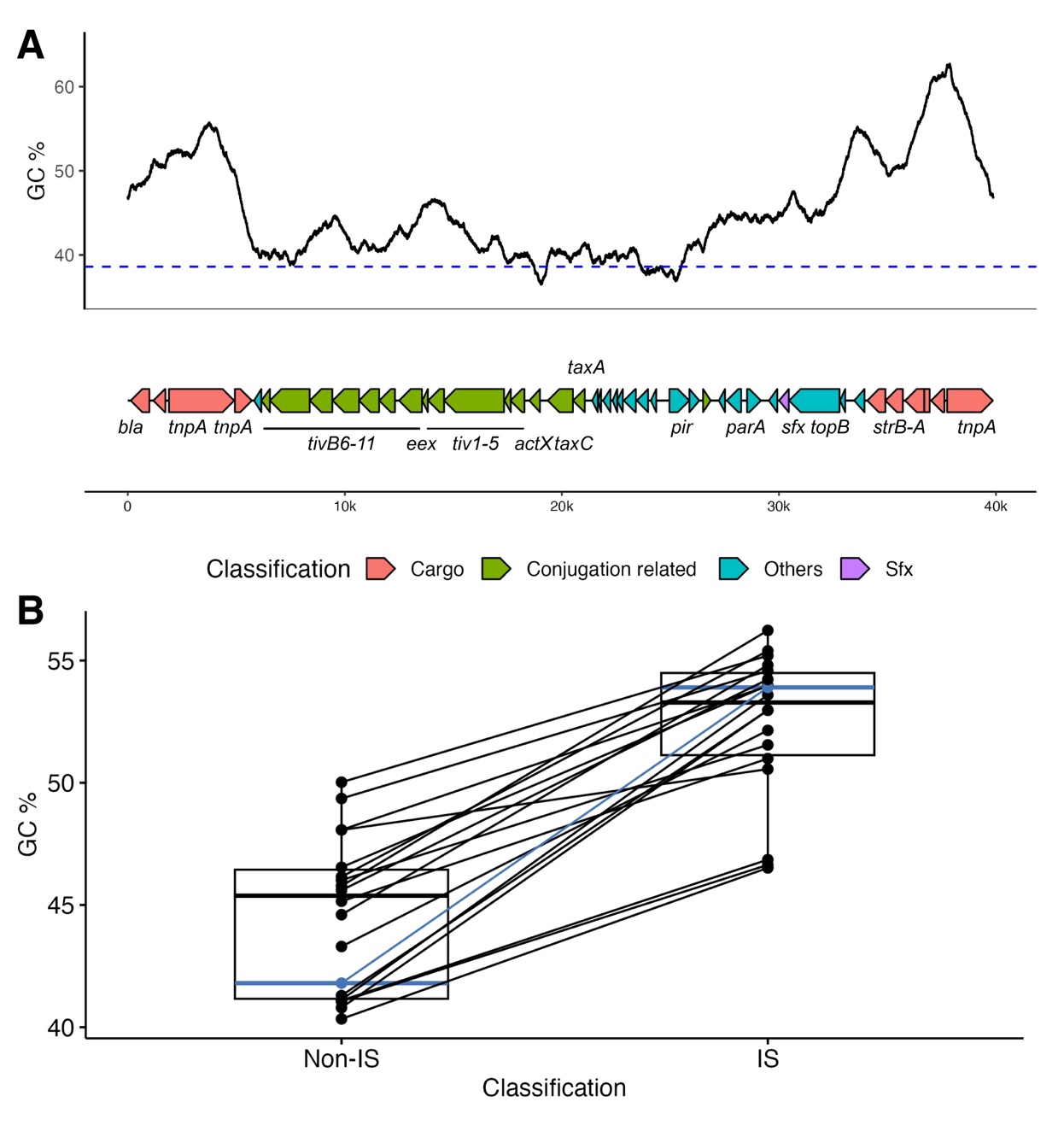


**Figure S5. IncX plasmids carrying Sfx homologs display an atypical base composition.** (A) R6K cargo genes have a higher GC% than core plasmid genes. The line plot displays the 2kbp sliding-window average GC% across the R6K sequence. The corresponding gene annotation derived from the RefSeq database (NCBI accession: NZ_LT827129.1) is shown below. Genes are colored based on functional categorization. The average GC% of H-NS binding sites (38.6% GC) is indicated by a blue dashed line. (B) IncX plasmids with Sfx homologs display an atypical base composition. The paired dot plot displays the GC% of non-insertion element (non-IS) and insertion element (IS) regions of 19 IncX plasmid representatives that carry a Sfx homolog. Each connected pair of points represents the value of one plasmid. The points representing R6K are colored in navy blue.
